# TiO_2_ Nanotube-Carbon (TNT-C) as Support for Pt-based Catalyst for High Methanol Oxidation Reaction in Direct Methanol Fuel Cell

**DOI:** 10.1186/s11671-016-1587-2

**Published:** 2016-12-28

**Authors:** M. Abdullah, S. K. Kamarudin, L. K. Shyuan

**Affiliations:** 1 Fuel Cell Institute, Universiti Kebangsaan Malaysia, 43600 UKM Bangi, Selangor Malaysia; 2 Department of Chemical and Process Engineering, Faculty of Engineering and Built Environment, Universiti Kebangsaan Malaysia, 43600 UKM Bangi, Selangor Malaysia

**Keywords:** TiO_2_ nanotubes, Pt-based catalyst, Methanol oxidation, Direct methanol fuel cell

## Abstract

In this study, TiO_2_ nanotubes (TNTs) were synthesized via a hydrothermal method using highly concentrated NaOH solutions varying from 6 to 12 M at 180 °C for 48 h. The effects of the NaOH concentration and the TNT crystal structure on the performance for methanol oxidation were investigated to determine the best catalyst support for Pt-based catalysts. The results showed that TNTs produced with 10 M NaOH exhibited a length and a diameter of 550 and 70 nm, respectively; these TNTs showed the best nanotube structure and were further used as catalyst supports for a Pt-based catalyst in a direct methanol fuel cell. The synthesized TNT and Pt-based catalysts were analysed by FESEM, TEM, BET, EDX, XRD and FTIR. The electrochemical performance of the catalysts was investigated using cyclic voltammetry (CV) and chronoamperometric (CA) analysis to further understand the methanol oxidation in the direct methanol fuel cell (DMFC). Finally, the result proves that Pt-Ru/TNT-C catalyst shows high performance in methanol oxidation as the highest current density achieved at 3.3 mA/cm^2^ (normalised by electrochemically active surface area) and high catalyst tolerance towards poisoning species was established.

## Background

Fuel cells are electrochemical devices that can convert chemical energy into electricity by means of an electrochemical reaction. Direct methanol fuel cells (DMFCs) are effective electrochemical devices that have been receiving considerable attention due to their ease of handling and operation for portable applications. Ultimately, there are two basic chemical reaction mechanisms involved in DMFCs, as follows: (i) methanol oxidation (anode side) and (ii) oxygen reduction (cathode side) reactions. In methanol oxidation, methanol is electro-oxidized into CO_2_, and in oxygen reduction, oxygen is reduced to water or steam. In DMFCs, the choice of catalyst used is the main factor in achieving high electrocatalytic activity.

1 $$ \mathrm{Anode}\kern-0.25em :\ {\mathrm{CH}}_3\mathrm{O}\mathrm{H} + {\mathrm{H}}_2\mathrm{O}\to {\mathrm{CO}}_2 + 6{\mathrm{H}}^{+} + 6{\mathrm{e}}^{-} $$

2 $$ \mathrm{Cathode}\kern-0.25em :\ 3/2{\mathrm{O}}_2 + 6{\mathrm{e}}^{-} + 6{\mathrm{H}}^{+}\to 3{\mathrm{H}}_2\mathrm{O} $$

In this study, the focus will be on the anode side, which is the site of the methanol oxidation reaction. Normally, Pt is a common catalyst used on the anode side, but the intermediate product of CO that is generated during the reaction makes it less favourable as an electrocatalyst. Moreover, CO that is strongly adsorbed onto the platinum active sites tends to worsen the performance by increasing the level of catalyst poisoning. As a common catalyst support in DMFC, carbon black suffers from corrosion and, at the same time, decreases the durability and stability of the catalyst itself. Therefore, discovering an alternative support for the main catalyst is required to solve this crucial problem. Dispersing the Pt-Ru catalyst onto the catalyst support is one of the main factors that lead to high electrocatalytic activity in the catalyst itself. The main properties that ultimately can increase the electrocatalytic activity of the catalyst will be high surface area that may be suitable for dispersion of the metal catalyst with even distribution. Moreover, porous structures will be a great idea as the surface will be high and good for moving electron and proton during the ion exchange process. A good catalyst support also should have strong metal support properties and have good electronic conductivity. Recently, the combination of Pt-based catalysts with metal oxide materials as the catalyst support (e.g. Pt/TiO_2_, Pt/CeO_2_, Pt/WO_3_) has been actively studied to discover the potential advantages of these combined materials for applications in direct methanol fuel cells [1–13]. Among these materials, Titania (TiO_2_) has high potential in this field as it offers a low manufacturing cost, good stability in electrochemical environments, high corrosion resistivity towards aqueous media, strong metal support interaction and good proton conductivity. However, Titania has low electronic conductivity compared to carbon black. Many considerable efforts had been made in order to improve the conductivity for catalyst support purposes. One of the famous ways to overcome this problem is by fabricating TiO_2_ in special nanostructure like nanotube nanosheet, nano-fibre, nanosphere and nano-ribbon [2–5, 14–18]. Titania in the form of nanotubes, also known as TiO_2_ nanotubes (TNTs), offers a good nanostructure for supporting catalysts as it has a high surface-to-volume ratio, which could enhance the dispersion of the main catalyst [19]. Moreover, the combination of TiO_2_ with conductive agent like carbon is also one of the great steps for improving the conductivity of TiO_2_ [20]. Hence, in this study, the TNTs were used as catalyst support together with carbon black for improving the catalytic activity of Pt-based catalyst for methanol electro-oxidation. Although carbon has some drawback as catalyst support, the high electronic conductivity of the carbon itself makes it interesting for catalyst support and needs to be improved by introducing the TNT as a co-support as it can provide a better place for dispersion of catalyst by having strong interaction with carbon and thus forming a three-phase interface on the surface of TNT. The carbon may act as a strong link for TNT and catalyst deposited which is Pt-Ru and makes it distributed evenly on the surface of support.

In general, TNTs can be synthesized by four common methods: (i) the hydrothermal method, (ii) the electrochemical anodization method, (iii) the template-assisted method and (iv) the sol-gel method. In this study, a hydrothermal method was used to produce TiO_2_ nanotubes; this method was environmentally friendly and economical, and it provided high-quality TNTs with a high surface area compared with the other methods. The main objective in this study was to determine the effect of highly concentrated NaOH on the structure of TNTs, and the best TNTs that were synthesized were further used as a catalyst support for a Pt-based electrocatalyst for methanol electro-oxidation in a DMFC. The TNTs were synthesized using the hydrothermal method proposed by Kasuga et al. [21, 22], which was followed by an acid washing process to remove sodium (Na) compounds. Pt-Ru nanoparticles were dispersed onto the TNT support using an impregnation method with H_2_PtCl_6_ and RuCl_3_ as the precursors. The prepared catalyst was characterized by field-emission scanning electron microscopy (FESEM), transmission electron microscopy (TEM), X-ray diffraction (XRD), Fourier transform infrared (FTIR) spectroscopy, energy-dispersive X-ray (EDX) and adsorption and desorption of N_2_ gas analysis. The electrocatalytic activity of the catalyst was analysed by cyclic voltammetry (CV) and chronoamperometric (CA) analysis.

## Methods

### Experimental

#### Chemicals/Materials

For the synthesis of TNTs, TiO_2_ nanopowder (P25, Degussa (99.5 %, 21 nm)) was purchased from Sigma-Aldrich, USA, and sodium hydroxide, NaOH, was purchased from Macron, USA. The HCl for the acid washing process was purchased from J.T Baker, USA. Hexachloroplatinic acid, H_2_PtCl_6_ (37.5 % Pt), ruthenium chloride, RuCl_3_ (45–55 %) and sodium borohydride, NaBH_4_ (96 %) were purchased from Sigma-Aldrich and used in the impregnation process of Pt nanoparticles into TNTs. For electrochemical performance testing, H_2_SO_4_ and CH_3_OH were purchased from Sigma-Aldrich, and the Ag/AgCl reference electrode was purchased from Metrohm.

### Synthesis of TiO_2_ Nanotubes

TNTs were synthesized using the hydrothermal method proposed by Kasuga et al. [22]. In a typical procedure, 3 g of TiO_2_ nanopowder was dispersed in 100 mL aqueous solution of NaOH that varied in concentration, including 6, 8, 10 and 12 M. The mixture was first ultrasonicate for half an hour, followed by stirring for 1 h to allow the solution to completely mix. Then, the mixture was transferred into a Teflon-lined stainless steel autoclave, and a hydrothermal treatment was performed for 48 h at 180 °C. After the alkaline treatment, the precipitate was collected and washed with deionized water, 0.1 M HCl until the pH was equal to 2 and deionized water again until the pH was equal to 7. The washing process must be performed properly to make sure that the sodium content is fully removed to ensure that the replacement of Na^+^ with H^+^ ions was performed properly. The sample was then filtered and dried overnight at 80 °C. The samples were labelled TNT-6, TNT-8, TNT-10 and TNT-12 according to the concentration of NaOH used, which are 6, 8, 10 and 12 M, respectively. Lastly, TNTs were obtained as a white product after drying and grinding to obtain the TNTs in powder form. The samples were then sent to characterize the surface morphology and crystallographic structure of the TNTs.

### Deposition of Pt-Ru Nanoparticles into TiO_2_ Nanotubes

The Pt-based nanoparticles were deposited into the TiO_2_ nanotubes referring to the chemical reduction method proposed by Chen et al. [6], Ito et al. [23] and Abida et al. [2, 5]. The reduction of Pt-Ru nanoparticles onto TNTs and carbon supports was obtained using NaBH_4_ as the reduction agent and H_2_PtCl_6_ and RuCl_3_ as the precursor both for Pt and Ru. First, the support material consisting of TNT and carbon black was ultrasonically dispersed in a mixture of distilled water and isopropyl alcohol (IPA) with ratio 50:50 for half an hour. Then, a 10 wt% solution of Pt-Ru (1:1) was acquired by adding H_2_PtCl_6_ and RuCl_3_ as the precursors into the support solution. The pH value of the mixture solution was adjusted until the pH was equal to 8, and the temperature was raised to 80 °C and maintained for several minutes. After that, 50 ml of 0.4 M sodium borohydride, NaBH_4_, was added drop by drop into the solution under vigorous stirring for 2 h. The catalyst was then cooled down and repeatedly washed with distilled water. Lastly, the catalyst was filtered and dried for 4 h at 110 °C.

### Characterization of Catalysts

The morphology and structure of the prepared catalyst were analysed using TEM and FESEM model of Tecnai G2 F20 X-Twin (FEI) and SUPRA 55VP, respectively. A sample was prepared for TEM analysis by mixing the catalytic powder with a certain amount of ethanol and continuing the ultrasonic treatment for several minutes. The catalytic solution with no impurities was then placed onto a copper grid. The percentage of element present in the chemical composition of the catalyst was determined using EDX, model FEI Quanta 400 FESEM.

The Brunauer-Emmett-Teller (BET) surface area of the prepared catalyst was determined from the nitrogen adsorption and desorption isotherms at 77 K using a Micromeritics ASAP 2020 instrument. The bonding structure of the catalyst was studied using Fourier transform infrared near infrared (FTIR-NIR), PerkinElmer model Spectrum 400 FT-IR and XRD analysis, model Bruker/D8 Advance.

The electrochemical analysis of the catalyst powder was performed by CV and CA using an Autolab potentiostat-galvanostat using a three-compartment electrode, including glassy carbon electrode (GCE) with 3 mm diameter (*A* = 0.071 cm^2^) as the working electrode, a Pt counter electrode and a Ag/AgCl reference electrode. For the methanol oxidation test, a solution of 1 M CH_3_OH and 0.5 M H_2_SO_4_ was used. For the preparation of electrode, the GCE was first polished with an Al_2_O_3_ solution and deionized (DI) water until a clear surface was obtained by a smooth and gentle cleaning method. Then, the GCE was washed by ultrasonication for 5 s to ensure that the surface of the electrode was completely clean. To deposit the catalyst, an ink was prepared by mixing 5 mg of the catalyst prepared with 80 μL isopropanol, 80 μL DI water and 50 μL 5 wt% Nafion solution. The mixing solution was then ultrasonicated for 1 h to allow for complete dissolution. Next, approximately 2.5 μL of the well-dissolved ink was deposited onto the GCE surface using a micro-pipette and allowed to dry. Nitrogen gas was bubbled into the supporting electrolyte before performing the electrochemical analysis. For the CV analysis, the test was recorded at a scan rate of 50 mV/s from 0 to 1.3 V versus Ag/AgCl, and 10 cycles were needed to stabilize the current-potential signal.

## Results and Discussion

### Synthesis of TiO_2_ Nanotubes

The TNTs were obtained by a hydrothermal method, which introduced a high concentration of NaOH and pure TiO_2_ powder. Several studies from established researchers [2, 3, 5] generally agree that the hydrothermal product can be X_2_Ti_2_O_5_·H_2_O in which *X* can be either be Na or H. The reaction step of the TNT formation can be defined by the chemical equation below.

(i) Reaction with high concentration of NaOH

In the hydrothermal reaction, the TiO_2_ powder reacts with a highly concentrated NaOH aqueous solution at 110 °C for 24 h. The product of this reaction is sodium titanate (Na_2_Ti_2_O_5_·H_2_O). The chemical reaction can be described as follows:

3 $$ 2{\mathrm{Ti}\mathrm{O}}_2 + 2\mathrm{NaOH}\to {\mathrm{Na}}_2{\mathrm{Ti}}_2{\mathrm{O}}_5\cdotp {\mathrm{H}}_2\mathrm{O} $$

(ii) Acid washing treatment

The sodium titanate (Na_2_Ti_2_O_5_) was thermodynamically unstable and may decompose into Na_4_Ti_5_O_12_ and Na_8_Ti_5_O_14_. The decomposition of the sodium titanate was prevented by washing the white slurry solution with DI water and followed by acid solution HCl until pH = 2 and repeated again with DI water until pH = 7. The ion exchange between Na^+^ and H^+^ occurs during the acid wash treatment, and hydrogen titanate forms after the process is complete. The general chemical reaction can be described as follows:

4 $$ {\mathrm{Na}}_2{\mathrm{Ti}}_2{\mathrm{O}}_5\cdotp {\mathrm{H}}_2\mathrm{O} + x\mathrm{H}+\to \kern0.5em \mathrm{H}x{\mathrm{Na}}_{2-x}{\mathrm{Ti}}_2{\mathrm{O}}_5\cdotp {\mathrm{H}}_2\mathrm{O} + x{\mathrm{Na}}^{+},\ \left(0\ \le\ x\ \le\ 2\right) $$

(iii) Dehydration process

The dehydration process is performed by drying the sample at 80 °C overnight. The reaction consists of two steps as follows:

5 $$ {\mathrm{H}}_2{\mathrm{Ti}}_2{\mathrm{O}}_5\cdotp {\mathrm{H}}_2\mathrm{O}\to {\mathrm{H}}_2{\mathrm{Ti}}_2{\mathrm{O}}_5 + {\mathrm{H}}_2\mathrm{O} $$

6 $$ {\mathrm{H}}_2{\mathrm{Ti}}_2{\mathrm{O}}_5\to 2{\mathrm{Ti}\mathrm{O}}_2 + {\mathrm{H}}_2\mathrm{O} $$

### Morphology of TNT

**Fig. 1:**
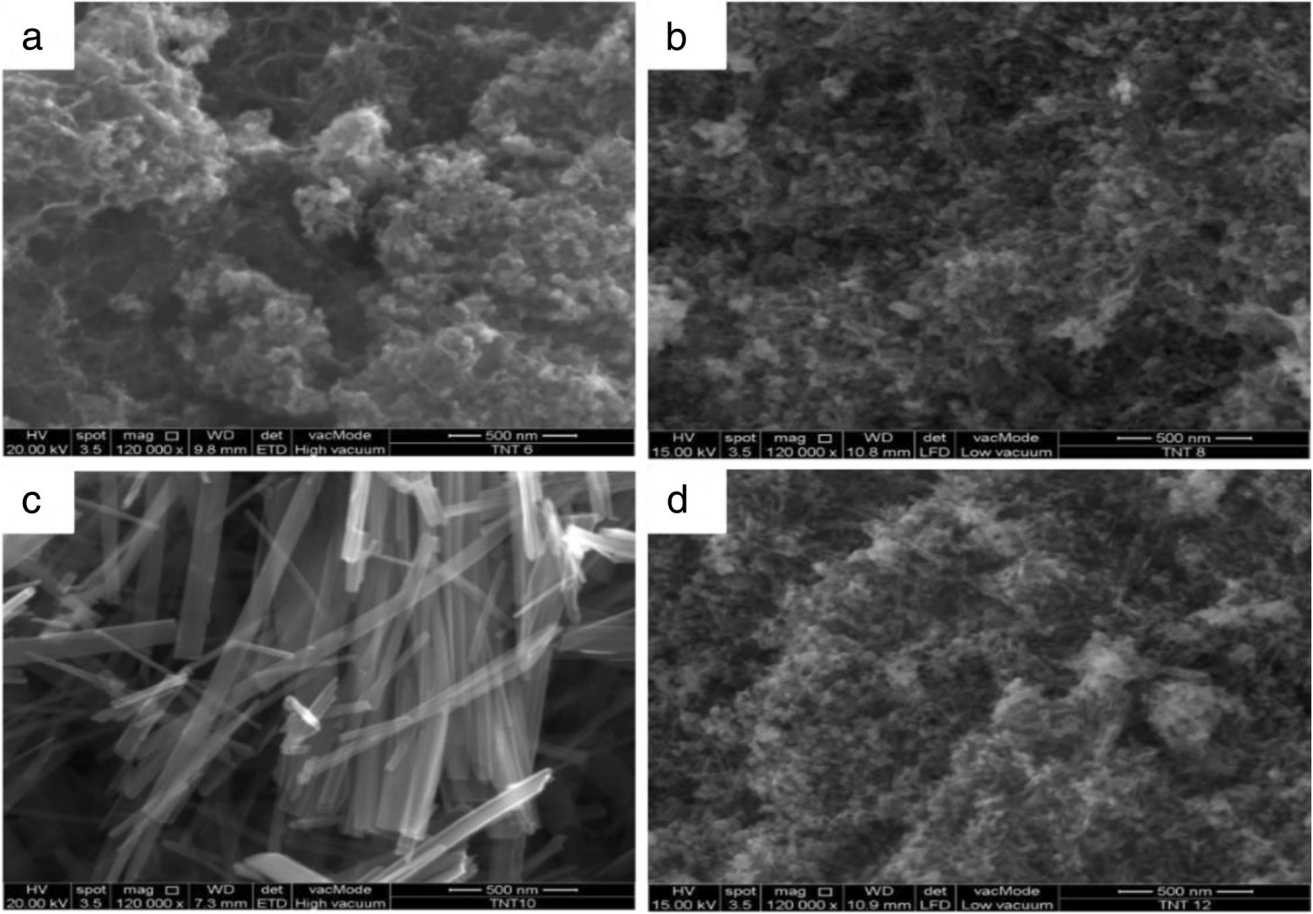
FESEM images of TiO_2_ nanotubes (TNT) based on different concentrations of NaOH: **a** 6, **b** 8, **c** 10 and **d** 12 M

**Fig. 2:**
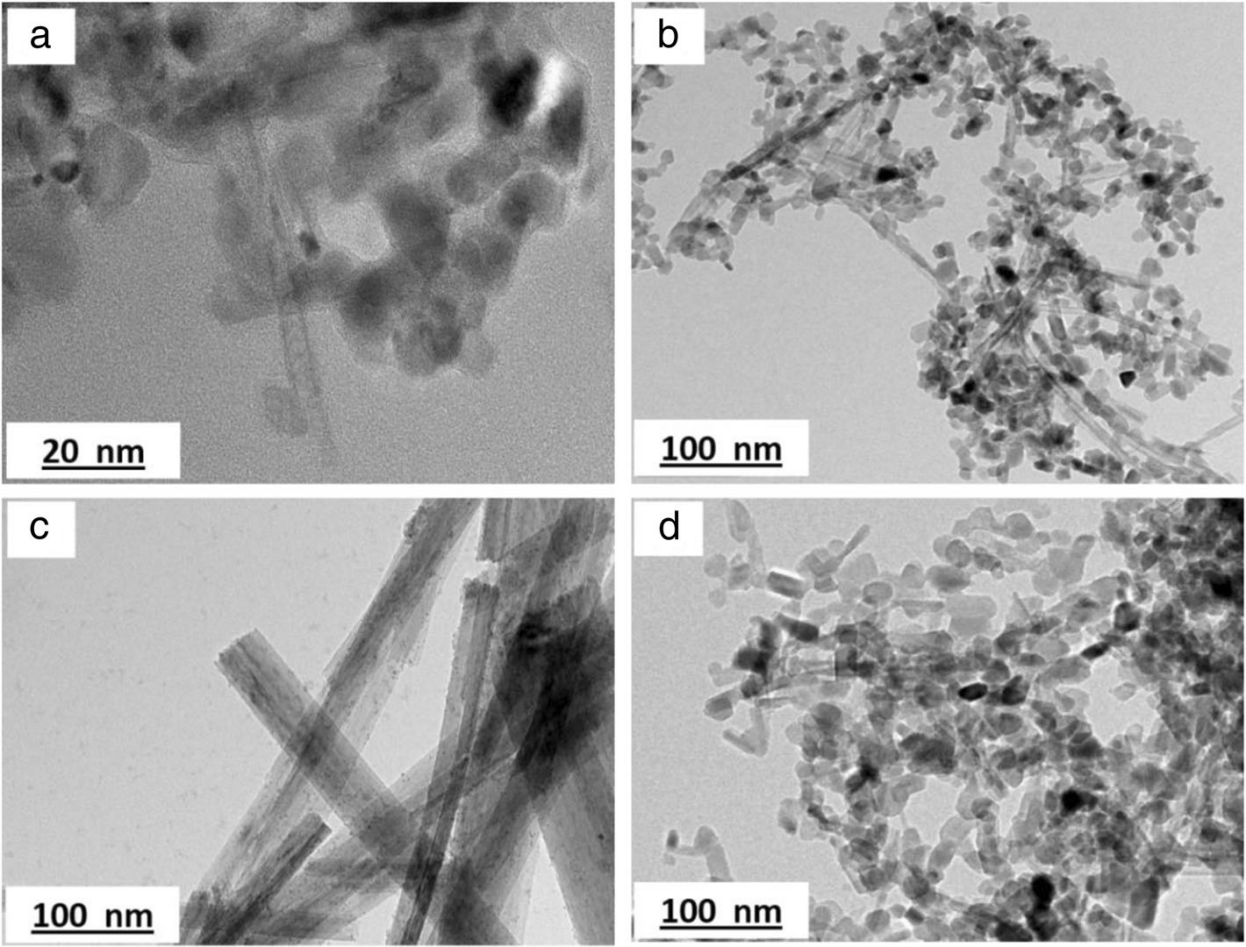
TEM images of TNTs prepared: **a** TNT-6, **b** TNT-8, **c** TNT-10 and **d** TNT-12 at scale of 20 nm for TNT-6 and 100 nm for others

**Fig. 3:**
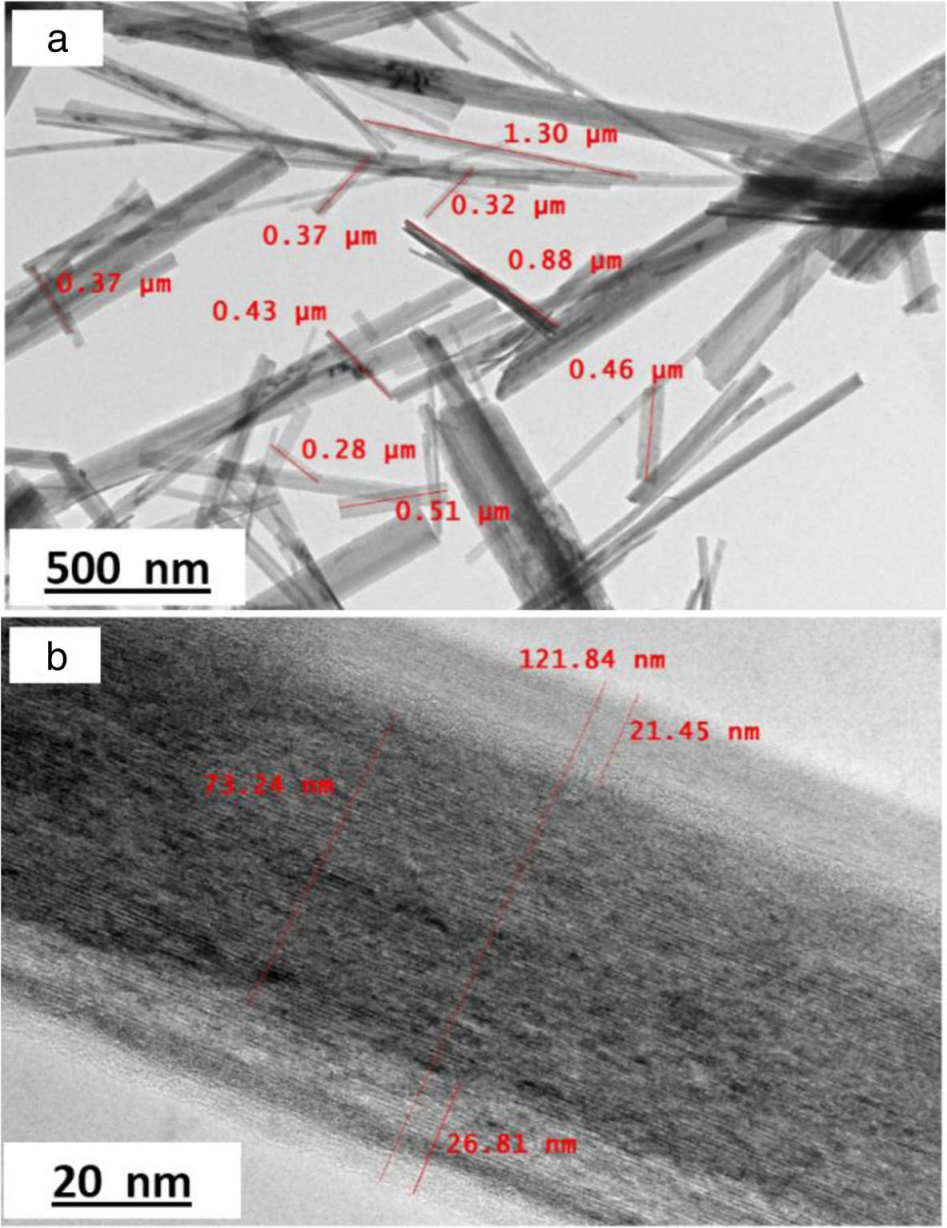
TEM images of TNT-10 with dimension measured (**a**, **b**)

The morphology structure of the TNTs formed with different concentrations of NaOH was analysed using FESEM and is presented in Fig. 1. Based on the figure, only Fig. 1c shows the complete nanotube structure of TiO_2_, which refers to the sample TNT-10, which used a NaOH concentration of 10 M. For TNT-6 in Fig. 1a, the nanostructure started to form with early tube-like formations, and according to Huang et al. [24], the structure would likely be the first stage of nanosheet formation due to the same synthesis conditions at lower alkaline conditions and this was proven by analysing TEM images for all samples in Fig. 2. The nanosheet for TNT-6 was seen in short-sheet, and it can only be viewed at very low scale which is 20 nm. However, TNT-8 in Fig. 1b has shown that the nanotube structure was starting to break down into separate but still incomplete tube structures. The non-complete structure of TNT-8 can be viewed clearly in Fig. 2b based on TEM analysis. Furthermore, for TNT-10, the nanotube structure was completely formed with a multi-layered structure. The TEM image in Fig. 2c has shown more clear structure of TNT with forming smooth structures with random alignment. However, in Fig. 1d, which is TNT-12, the nanotube structures have almost ruptured because the concentration of NaOH introduced during the hydrothermal treatment was too high [25]. The nanostructures that may exist for TNT-12 would be amorphous nanoparticles as in Fig. 2d which does not show any tube formation. Moreover, many aggregations and unreacted compounds were found for both TNT-6 and TNT-12 with no tubular structure for the same reason. The increase in the diameter and length of the TNTs was detected with the increasing concentration of NaOH. For TNT-8, the mixture of nanoparticles and nanotube structures that started to form can be clearly observed, while for TNT-10, the tube structure was formed completely after the dehydration process. The nanotube structures for both TNT-8 and TNT-10 were formed as multi-layered nanostructures and existed in random alignment. Figure 3 shows the TEM image of TNTs for TNT-10 with dimension measured. An average length of 550 nm and a diameter of 70 nm for TNT-10 were obtained.

**Fig. 4:**
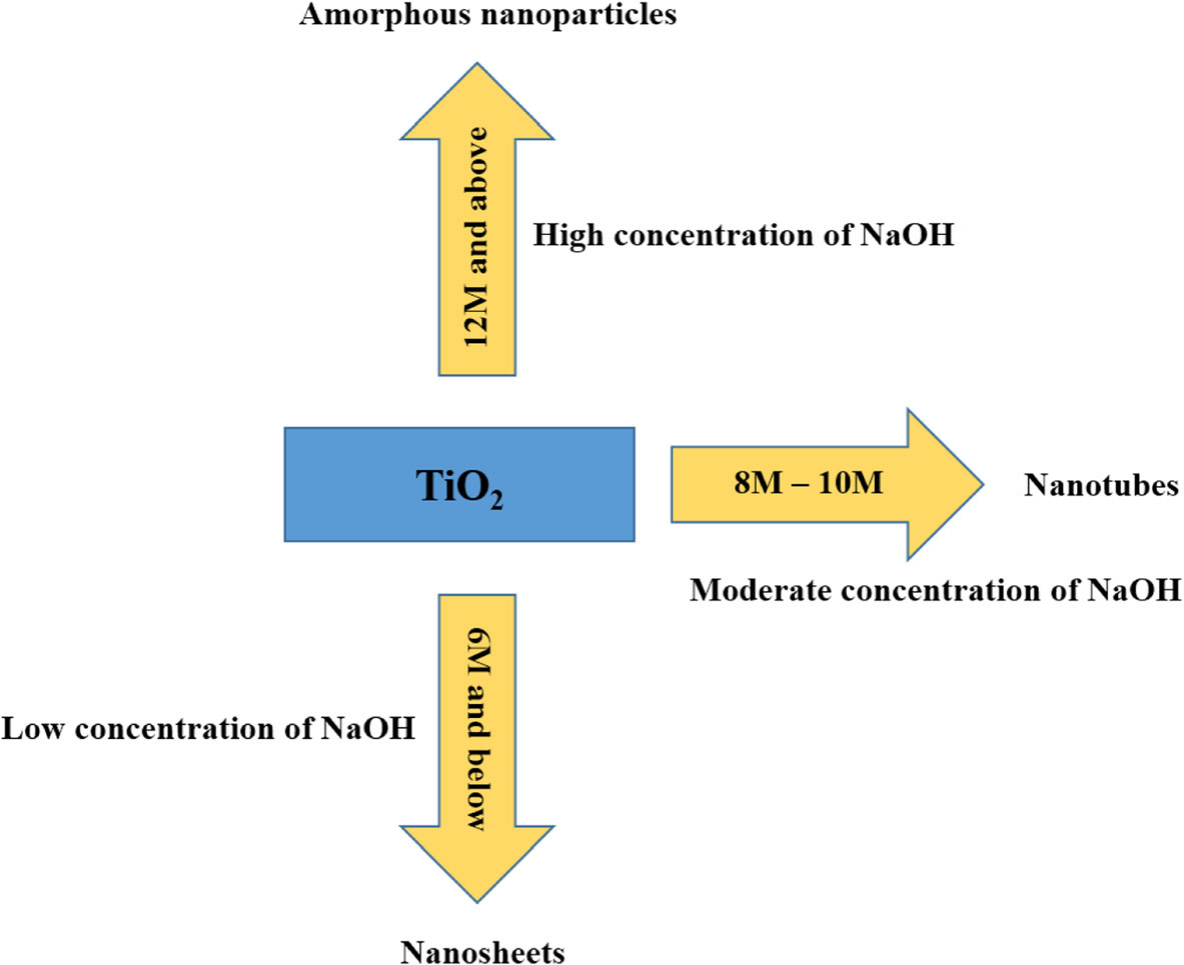
Schematic formation of TNTs based on different concentrations of NaOH during hydrothermal reaction

The formation of TNTs based on these synthesis results can be illustrated by the scheme in Fig. 4. TiO_2_ synthesized in different NaOH media could lead to the formation of various nanostructures. Lower alkaline conditions lead to the formation of nanosheet, while moderately alkaline solutions (8–10 M) result in nanotube formation. For the lower alkaline condition, the nanosheet was formed instead of nanotubes due to the rate of Ti ion diffusion that was too low and makes it unable to do the rolling process for tube formation [24]. Moreover, when low concentration of OH− ions was introduced in hydrothermal reaction, the shorter Ti–O bond will remained constant and unvaried at certain time and the nanosheet will stop rolling [26]. For higher concentrations of NaOH solution up to 12 M, the structure can move towards amorphous nanoparticles. At this time, the high alkaline condition tends to be very viscous and thus limited the diffusion of Ti (IV) ion due to the dissolution of the Titania precursor. Moreover, the crystallite Titania formed are nevertheless in an unstable state since it exists in extreme condition and, of course, the decomposition rate of crystallite Titania was faster compared to lower alkaline condition.

Besides, the rates of formation of hydrothermal product also depend on the rate of diffusion while diffusion depends on the concentration of dissolved Ti (IV) [24]. The higher increment of alkaline concentration for hydrothermal method, the faster and furious the dissolution rate of TiO_2_, and at the same time, the mass transfer in the reaction was also in good state. Therefore, the concentration of NaOH is an important parameter in producing TNTs as neither too much nor too little NaOH facilitates the rolling process of TiO_2_ nanosheet into nanotubes. Under 8-M NaOH, the nanotubes were starting to form, but the nanotube formation reached its limit at 10 M by completing the nanotube structures.

### Physical Characterization of TiO_2_ Nanotubes

**Fig. 5:**
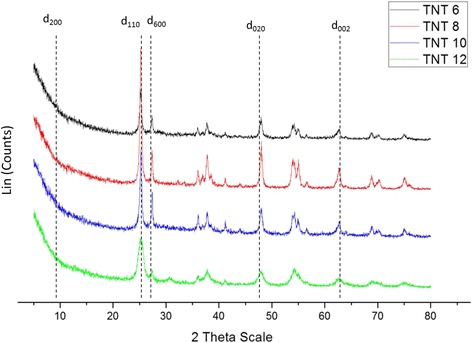
XRD pattern for TNT-6, TNT-8, TNT-10 and TNT-12

To discover the phase and composition of the prepared catalysts, XRD analyses were performed. Figure 5 shows the XRD patterns for four types of TNT products, which are TNT-6, TNT-8, TNT-10 and TNT-12. The XRD patterns for TNT-6 and TNT-12 exhibit almost the same pattern, but for TNT-8 and TNT-10, the XRD patterns were quite different. For TNT-8 and TNT-10, the anatase peaks of the TNT phase with a broad reflection peak were detected at 2*θ* = 25°, 37° and 47° and the rutile phase peaks were found at 2*θ* = 27°, 36° and 41°. Therefore, there was a mixture of both the rutile and anatase phases in the TNT composition. This is very interesting as the rutile phase is also discovered at low temperature synthesis and makes the TNT become more stable. The synthesis condition was one of the factors that make the rutile phase exists as the hydrothermal reaction was performed in a very close-tight reactor that could gave high pressure. The TNT-8 sample shows the highest intensity peak at approximately *d*_110_ (25°), which is the strong peak of TNTs. The XRD pattern of TNT-8 strongly indicated that it was similar to the hydrogenotitanate, H_2_Ti_2_O_5_·H_2_O, that was formed during the hydrothermal treatment. The intense peak at *d*_110_ (25°) indicates the interlayer spacing of the tetragonal system with lattice constants of *a*_o_ = 3.872 and *c*_o_ = 9.616. Moreover, the intense peak at *d*_110_ (25°) also decreases when approaching the *d*_600_ peak (27°). This proves that there was a loss of water molecules during the dehydration process. The crystallite sizes for TNT-6, TNT-8, TNT-10 and TNT-12 were 14.02, 14.37, 13.7 and 81.4 nm, respectively. The Na^+^ content can be determined from the relative ratio of the *d*_110_ to *d*_600_ peaks [3]. The ratio of *d*_110_:*d*_600_ seemed to increase as the values of theta increased, which indicated that the Na^+^ content decreased because of the replacement of Na^+^ by H^+^ that occurred during the acid washing treatment after the hydrothermal treatment [5].

**Fig. 6:**
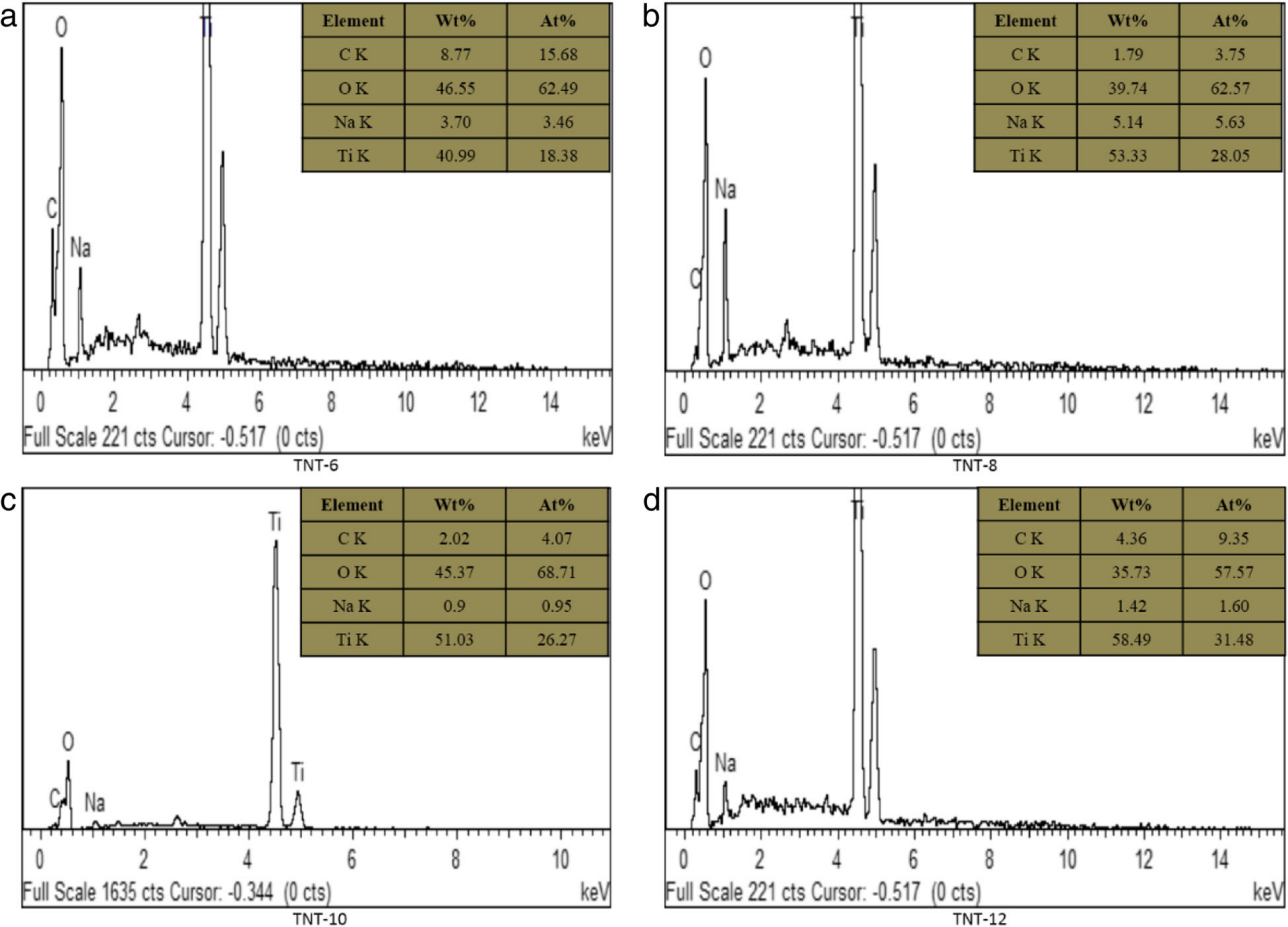
EDX pattern of TNTs prepared by different concentrations of NaOH (**a**–**d**)

The EDX patterns for TNTs prepared at 6, 8, 10 and 12 M are shown in Fig. 6. The values of Ti, O and Na were calculated using integration. The values for the C atoms that were present during the analysis are due to the carbon paper that was used as the substrate for the deposited TNTs and can be neglected. The atomic ratio of Na to Ti determined the degree of Na^+^ replacement by H^+^ during the acid washing process by HCl and is tabulated in Table 1. From these results, we can see that the atomic ratio of TNT-10 was the lowest among the four types of TNTs produced. The results suggest that the lower the content of Na^+^, the higher the degree of replacement by H^+^ during the acid soaking process, which increases the process of TiO_2_ nanosheet rolling to become TNTs. This result was compatible with the FESEM and XRD results, as discussed above as the TNT-10 shown the greatest and complete formation of tubular TiO_2_ nanostructures and the presence of TNT phase was highly detected compared to another samples. For TNT-12, even though the atomic ratio of Na to Ti was comparatively low, the nanotube structure cannot be observed in the FESEM image. For TNT-6 and TNT-8, the atomic ratio was slightly higher than the others, which indicates that not all Na^+^ was replaced by H^+^ during the acid washing process. The ratio of O to Ti was also calculated to determine the amount of titanate that was formed in the TNTs. According to Tacchini et al. [27], the titanate structure can be confirmed when the ratio of O to Ti is greater or equal to 2. The ratio of O to Ti implies the adsorption of water molecules onto the surface of TNT. For TNT-6, the ratio gives the highest value, which was much higher than 2. For TNT-8 and TNT-10, the value O to Ti ratio was in the range that showed titanate formation in the sample based on XRD, FESEM and TEM analyses. Only TNT-12 had a value for this ratio that was less than 2, which was 1.83. This value demonstrated that there was no formation of nanotubes in TNT-12, and this result agrees with the FESEM and XRD analyses regarding the nanotube structure.

**Table 1:** Ratio of Na/Ti and O/Ti for TNTs based on EDX

Sample	Na/Ti	O/Ti
TNT-6	0.19	3.4
TNT-8	0.20	2.23
TNT-10	0.036	2.62
TNT-12	0.051	1.83

**Fig. 7:**
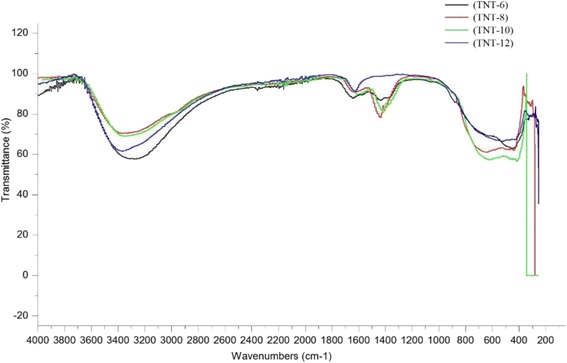
FTIR spectra for TNTs based on different NaOH concentrations

**Fig. 8:**
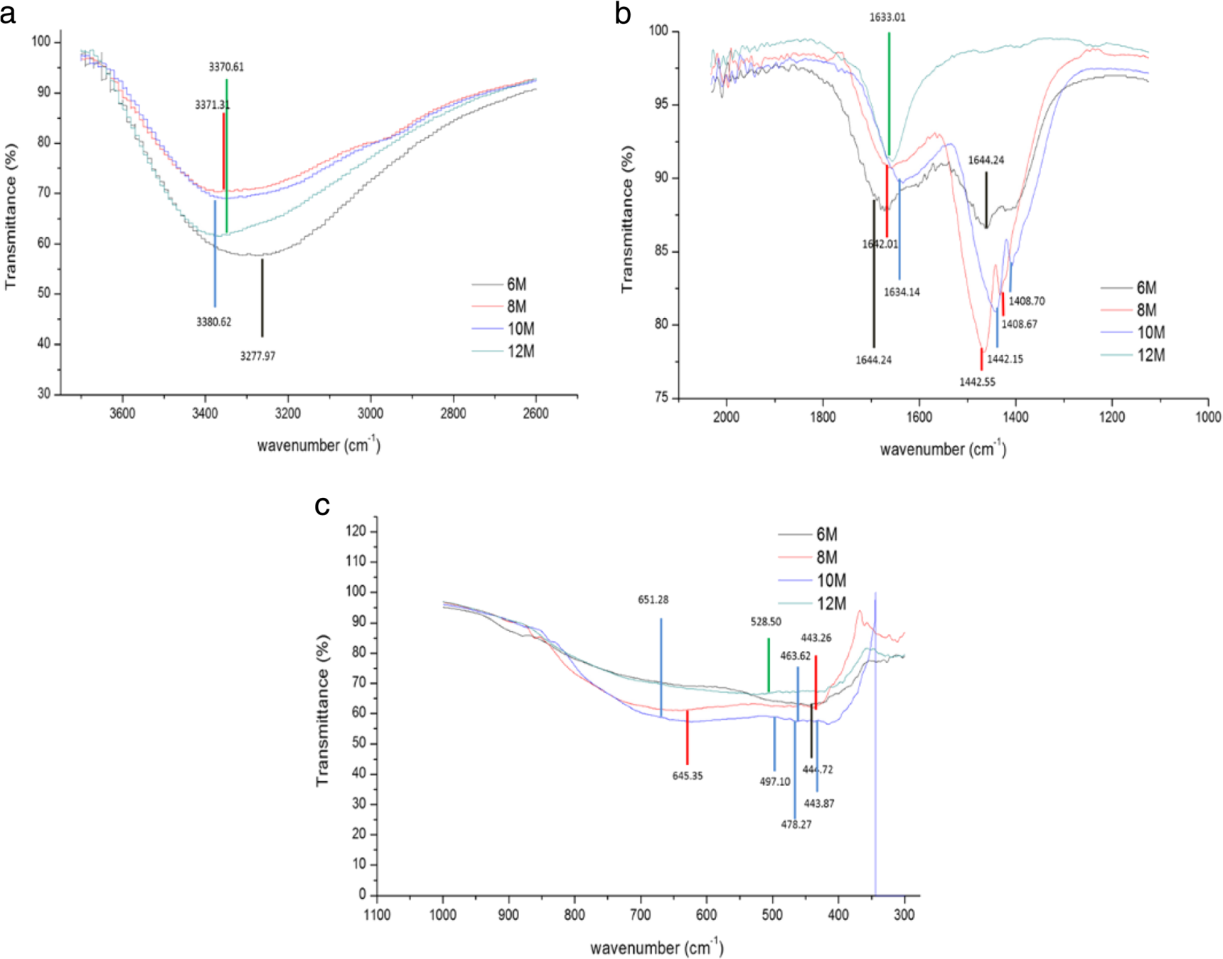
FTIR spectra until complete formation of TNTs: **a** OH group (3600–3200 cm^−1^), **b** Ti-OH group (1500–1100 cm^−1^) and **c** Ti-O group (700–400 cm^−1^)

The presence of chemical bonding in the TNT structure was examined using FTIR analysis in the wavenumber range of 4000 to 400 cm^−1^. Figure 7 presents the FTIR spectra for all types of TNT produced. The TNT formation can be explained by the FTIR spectra based on the changing peak generated during the analysis. The strong, sharp and broad intensity peak was discovered at wave numbers from 3200 to 3400 cm^−1^, which ensured that there was an O–H bond (hydroxyl group) present for all NaOH concentrations [28]. This peak in Fig. 8a implies the first stage of TNT formation, in which the hydrothermal reaction occurred. The NaOH reacted with TiO_2_ nanoparticles and formed sodium titanate. This result also shows that there were large amounts of O–H groups on the surface of the TNTs due to the stretching mode of Ti–OH bond. The sample TNT-10 exhibits the strongest vibrational modes for OH groups. Moreover, in Fig. 8b, strong peaks were also discovered from 1400 to 1600 cm^−1^, which also correspond to the O–H groups found on the TNTs bond that were present [29]. Moreover, the strong interaction between the Ti ions and the hydroxyl groups was due to the defect structure of the TNTs generated by oxygen vacancies [30]. This phenomenon can help the adsorption of water molecules onto the TNT surface and at the same time dissociate water molecules into OH– ions and H^+^. Due to this mechanism, a charged surface was present on the TNTs, depending on the protons present and the completion of the reaction of the surface hydroxyl groups. The discovery of TNT bonds was also because the Ti–O planes rolled into nanotubes and generated Brønsted acid sites and, at the same time, rapidly modified the Lewis acid site [31]. Pure TiO_2_, which is in either the anatase, rutile or brookite phase, contains Lewis acid sites, but after the introduction of concentrated NaOH into the pure TiO_2_, the Brønsted acid sites were generated. As the number of Brønsted acid site increases, better TNT structures can be achieved. The large amount of O–H groups in the TNTs can be enhanced for the purpose of depositing metal particles, which ease the process of dispersing and adhering the particles onto the nanotubes surface. In the region of approximately 700 to 400 cm^−1^ in Fig. 8c, a medium peak of Ti–O was found, which clearly suggested that the TNTs were completely formed after the acid washing process. Overall, TNT-10 showed the best results, as it had the highest peak intensity for both types of functional groups, which are hydroxyl groups and TNTs. The results strongly suggest that the formation of TNTs was influenced by the concentration of NaOH used during the hydrothermal reaction.

**Fig. 9:**
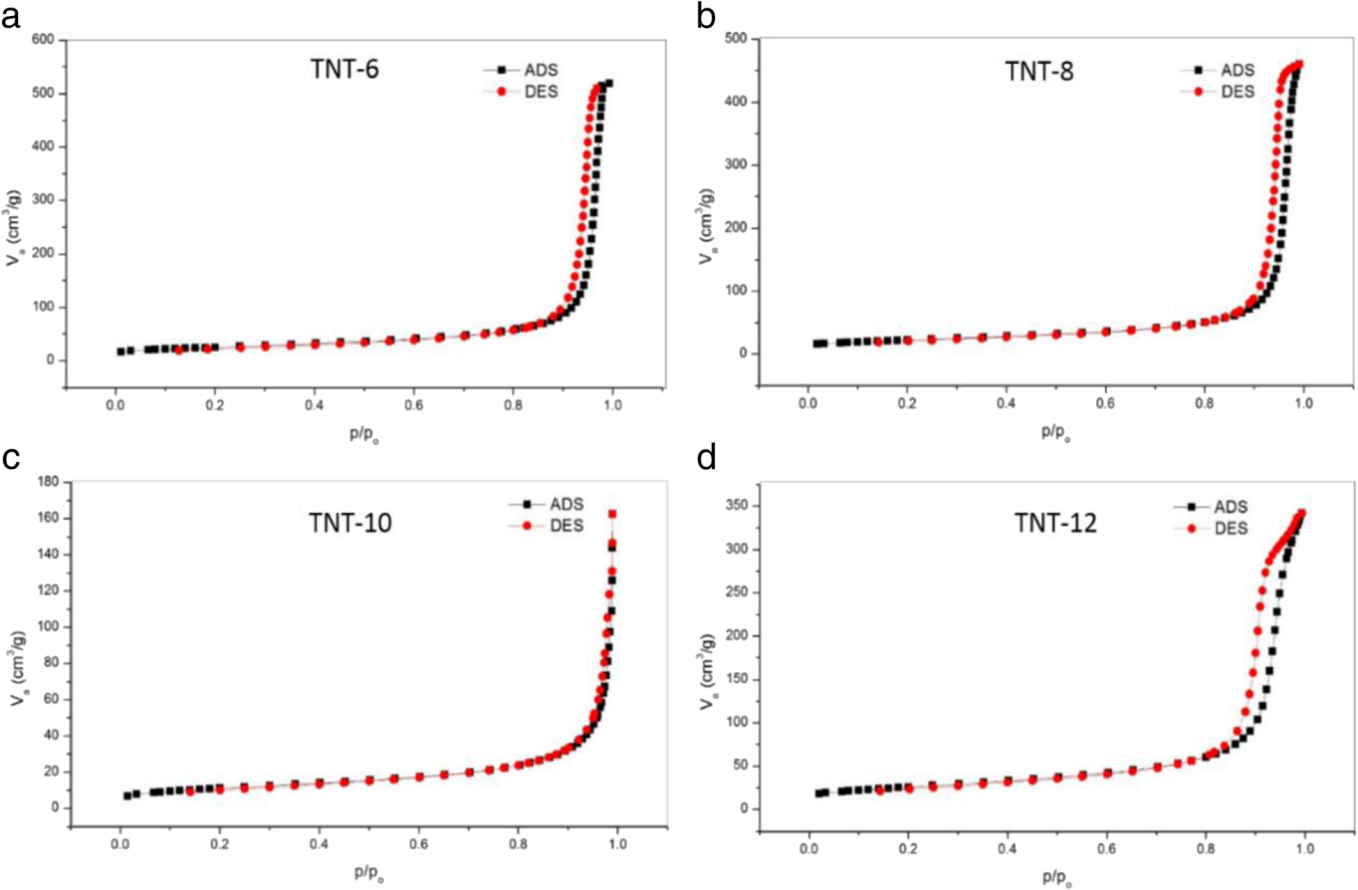
Adsorption (ADS)/desorption (DES) of N_2_ isotherm of TNTs at 77 K (**a**–**d**)

The adsorption and desorption of N_2_ gas was performed on the catalyst using the BET method to analyse the pore size distribution, mesoporous volume and surface area. The adsorption isotherm was obtained by measuring the amount of gas adsorbed onto the sample at relative pressures and constant temperature, normally 77 K, while desorption isotherm was measured when the gas was removed while decreasing the pressure. Figure 9 shows the nitrogen adsorption and desorption isotherm at 77 K for all TNT-based samples with various NaOH concentrations. The graph shows that the adsorption and desorption of N_2_ is type IV based on the IUPAC classification because all the TNT samples consist of mostly mesopores with pores in the range of 2–50 nm. The results from this analysis are tabulated in Table 2. Based on the results, TNT-12 showed the highest BET surface area of 92 m^2^/g compared with the other types of TNTs produced, which is in good agreement with Lee et al. [32], who stated that TNT synthesis at 180 °C gave the highest specific surface area. However, the BET surface area of TNT-10 showed the lowest surface area compared with the other three samples. This largest surface area was very important for the nanotube structure, as it provided an excellent space for the dispersion of the catalyst during the deposition of catalyst. The good dispersion of the catalyst plays an important role in increasing the performance of methanol oxidation, as it increases the metal support interaction between the catalyst support and catalyst. Although the BET area for TNT-12 was the highest among the samples, the area does not reflect the TNT area, as the tube formation was not discovered during the FESEM, XRD and FTIR analyses.

**Table 2:** Textural properties of all TNTs based on concentration (N_2_ adsorption/desorption)

Sample	*S*_BET_ (m^2^/g)	*V*_Total_ (cm^3^/g)	*D*_p_ (nm)
TNT-6	88	0.76	34.5
TNT-8	78	0.64	32.8
TNT-10	40	0.15	15.10
TNT-12	92	0.50	22.10
TiO_2_ (P25)	39	0.13	13

*S*_*BET*_ specific surface area, *V*_*Total*_ total pore volume, *Dp* average pore diameter

The above analyses of the synthesis of TNTs show that TNT-10, which used 10 M NaOH, showed excellent results in terms of structure, phase and composition with complete nanotube structures. Thus, TNT-10 was used in the next synthesis for the deposition of the Pt-based catalyst onto the TNT surface to analyse the electrocatalytic performance in methanol oxidation, which was measured by CV and CA analysis.

### Deposition Pt-based Catalyst onto Surface of TiO_2_ Nanotubes

#### Morphology of Deposited Catalyst

**Fig. 10:**
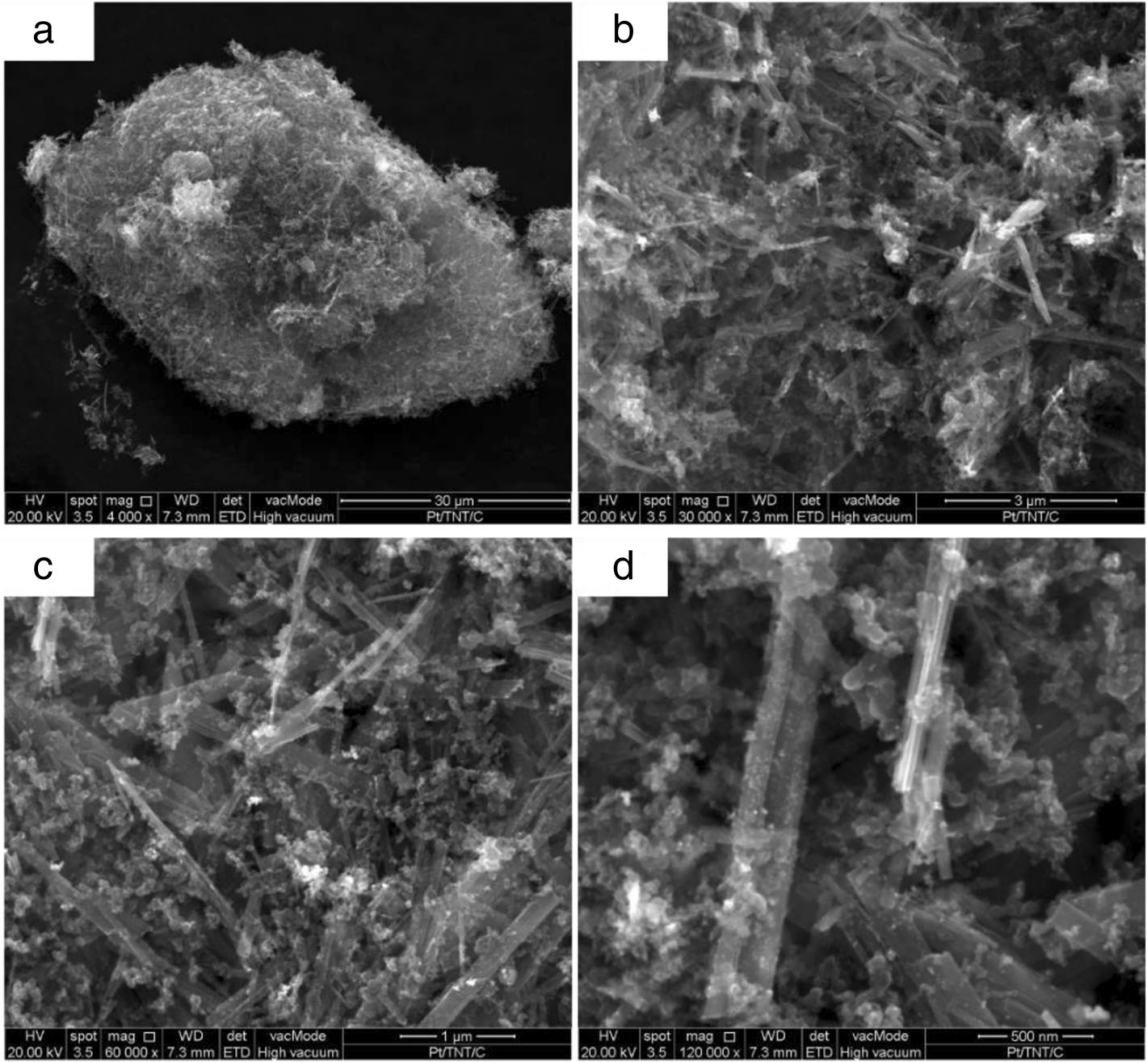
FESEM images of Pt/TNT/C catalyst at scale of **a** 30, **b** 3, **c** 1 and **d** 500 nm

**Fig. 11:**
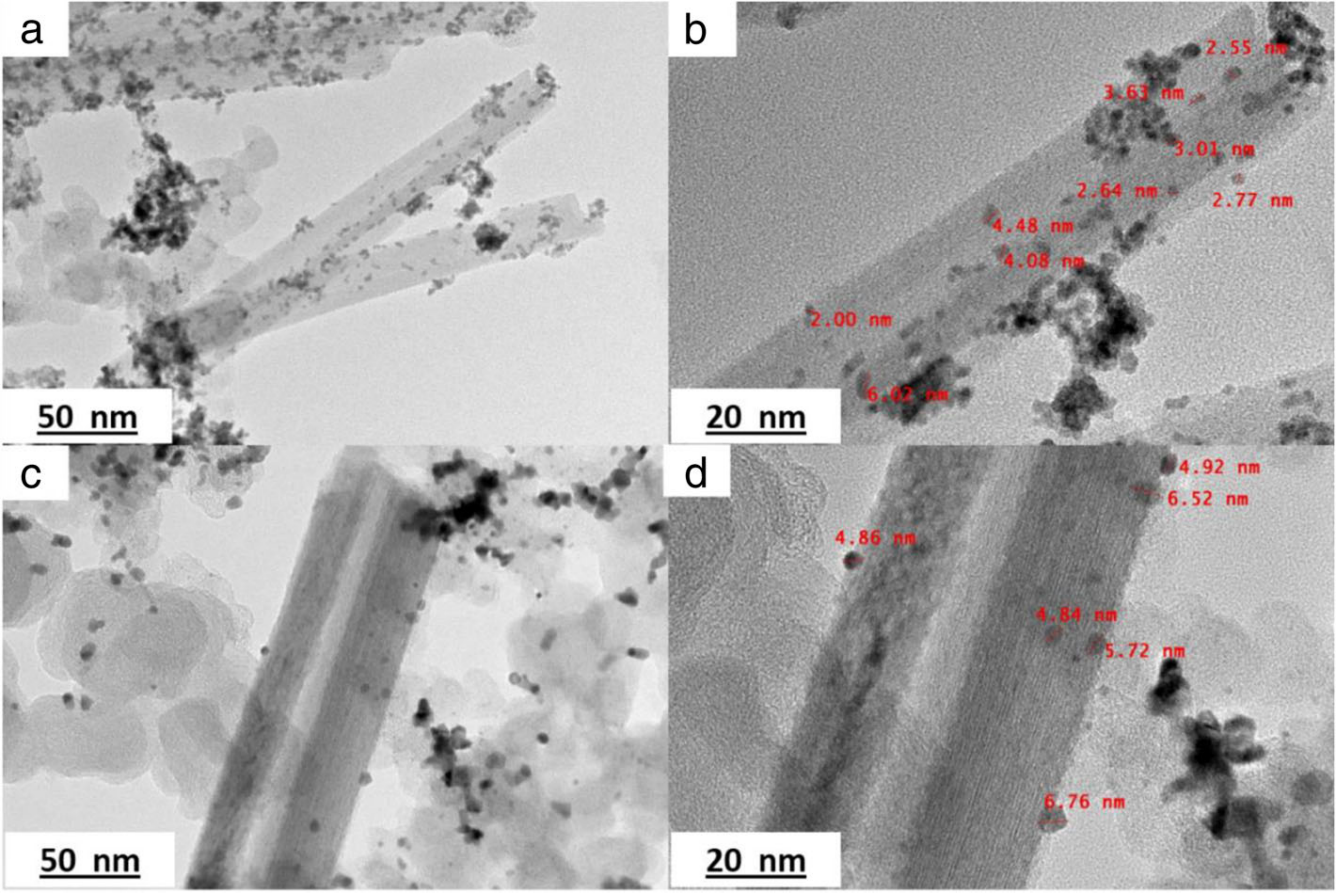
**a**–**d** TEM images of catalyst: [A]—Pt-Ru/TNT-C and [B]—Pt/TNT-C

The Pt-based catalyst onto TNT support was deposited using a chemical reduction method with H_2_PtCl_6_ for Pt/TNT and RuCl_3_ for Pt-Ru/TNT/C as the precursors. The FESEM image in Fig. 10 shows the Pt-based catalyst that was deposited onto the TNT surface. The catalyst adhered to the surface of the TNTs due to the strong metal support interaction between the catalyst and the TNTs. The strong metal support interaction can also be attributed to the high surface area of TNTs, which could enhance the dispersion of the catalyst in the tube structure. The TEM analysis was performed to examine the presence of the catalyst in the tube structure because inside the tube structure, there could be more space for the catalyst to disperse well, thus leading to a stronger support metal interaction. The TEM images in Fig. 11 shown the nanotubular structure of Pt-Ru/TNT/C and Pt/TNT/C, on which the catalysts were successfully deposited using the impregnation method. The metal catalysts were finely and uniformly dispersed, with an average size of 3.46 nm for a—Pt-Ru/TNT-C, and 4.83 nm for b—Pt/TNT-C. The size of metal was different for both samples because of the presence of the Ru for sample A due to the fact the Ru metal may enter the Pt face-centred cubic (f.c.c.) phase to form Pt-Ru alloys or another word halfly existed as oxide species. This situation can be explained more during the XRD analysis since the peak for Ru metal was detected almost at the same as Pt.

### Physical Characterization of the Deposited Catalyst

**Fig. 12:**
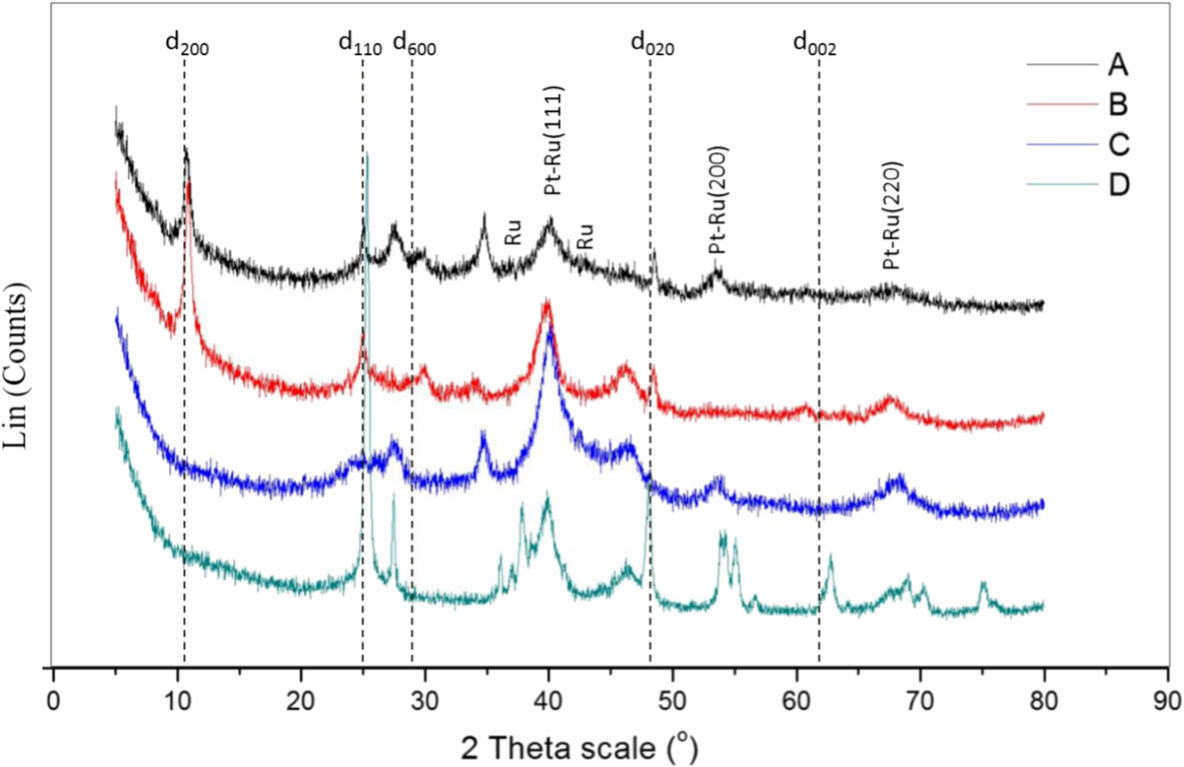
XRD patterns of catalyst: [A]—Pt-Ru/TNT-C, [B]—Pt/TNT-C, [C]—Pt/Ru-C and [D]—Pt/TiO_2_-C

Figure 12 shows the XRD patterns of Pt/Ru/TNT, Pt/TNT, Pt/Ru and Pt/TiO_2_, which are labelled as [A], [B], [C] and [D], respectively. For Pt/TiO_2_ (Degussa), the anatase peak was discovered at 2*θ* = 25.4° (*d*_101_) and the rutile peak at 2*θ* = 27.5° (*d*_110_). The crystallite size of the sample can be calculated from the X-ray spectral peaks of the anatase phase using the Scherrer formula:

7 $$ L=\frac{K\lambda }{\beta\ \cos\ \theta }, $$

where *L* = the crystallite size, *K* = 0.94, *λ* = the wavelength of X-ray radiation and *β* = the line width at half maximum height. From this equation, the crystallite size is approximately 25.02 nm. The Pt-Ru was detected at 2*θ* = 39.5^o^, 54^o^ and 68^o^ with lattice constant of *a* = 5.585, *b* = 5.585 and *c* = 5.585. The characteristic XRD peaks of platinum, Pt-Ru [(111), (200) and (220)] indicated that the metal existed as the f.c.c. phase. Furthermore, it can be implying that Ru metal may be included in the f.c.c. of Pt itself and forming Pt-Ru alloys as single phase structures and also lowering the lattice constant for Pt. The Ru peak was detected in the XRD spectra at 2*θ* = 37^o^ and 41.5^o^ with lattice constant, *a* = 4.48592, *b* = 4.48592 and *c* = 4.48592.

Compared with the Pt/TiO_2_ catalyst, the XRD patterns for Pt/Ru/TNT/C and Pt/TNT/C exhibit different peaks indicating the H_2_Ti_2_O_5_.H_2_O (hydrogenotitanate) phase, as the peaks at 2*θ* = 10.1°, 25°, 30° and 48° were firmly believed to be the TNT phase. The hydrogenotitanate was formed after the acid washing process, which means that the replacement of Na^+^ occurred at that time, and the ions were completely removed. The peaks showed an orthorhombic system with a lattice constant of *a*_0_ = 4.83780, *b*_0_ = 9.42080 and *c*_0_ = 2.95950. The characteristic Pt peaks were indexed to the f.c.c. phase [Pt (111), Pt (200) and Pt (220)].

**Fig. 13:**
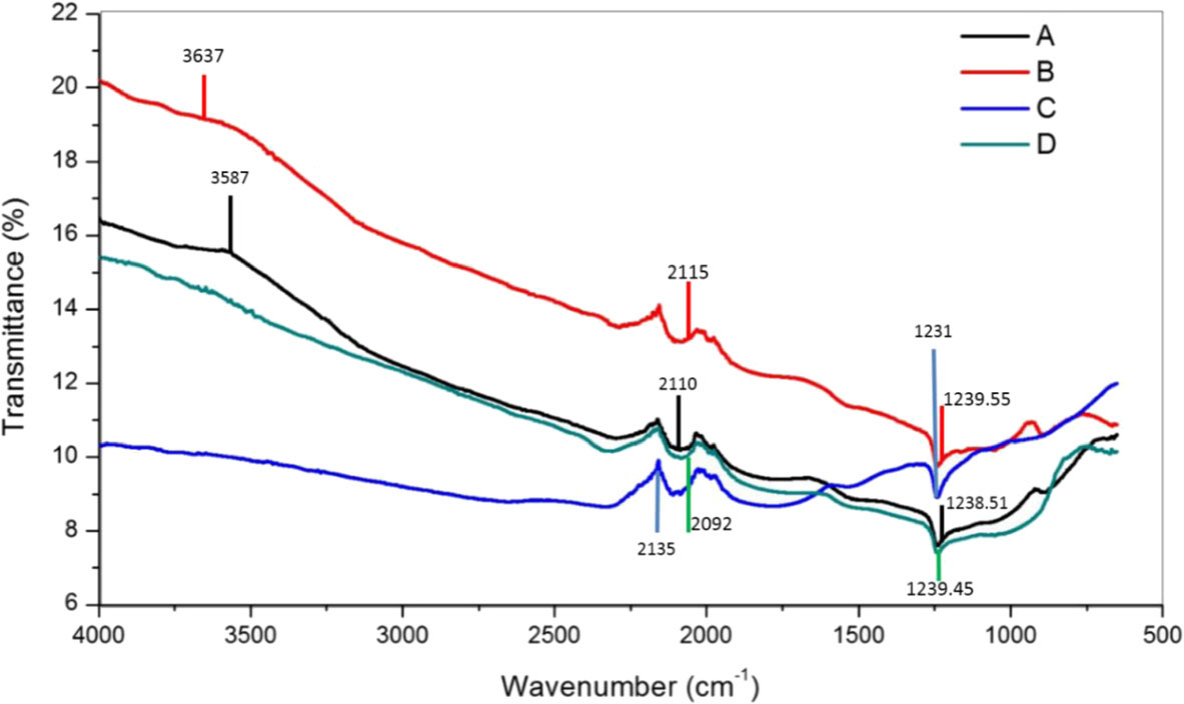
FTIR spectra of catalyst: [A]—Pt/Ru/TNT-C, [B]—Pt/TNT-C, [C]—Pt-Ru/C and [D]—Pt/TiO_2_-C

The chemical bonding was analysed by FTIR, and the results are presented in Fig. 13, with the intensities located at wave numbers from 650 to 4000 cm^−1^. The stretching vibration mode can be detected at 1238.51, 1239.55, 1231 and 1239.45 cm^−1^ for samples [A], [B], [C] and [D], respectively, which are attributed to the aliphatic amines (C–N) functional group in the medium stretching mode. An O–H functional group was detected at 3587 and 3637 cm^−1^ for samples [A] and [B], respectively, each showing strong and sharp vibration modes. Thus, the results can be attributed to the TNT bonds. The presence of O–H groups ensured that there were large amounts of O–H molecules on the TNT surface due to the stretching mode of the Ti–O bonds. For samples [C] and [D], the stretching mode was not detected from approximately 3600 to 3200 cm^−1^, which showed that there were TNTs present for that catalyst. Thus, the results were attributed to the catalyst with no TNTs as a support, which are Pt/Ru and Pt/TiO_2_. The carbon–carbon bond stretching (i.e. triple-bonded carbon) was also detected at 2110, 2115, 2135 and 2092 cm^−1^ for samples [A], [B], [C] and [D], respectively. This was due to the presence of carbon compounds in all the catalysts as the catalyst support.

### Electrochemical Performance of the Hybrid Catalyst for DMFC Application

#### Cyclic Voltammetry

**Fig. 14:**
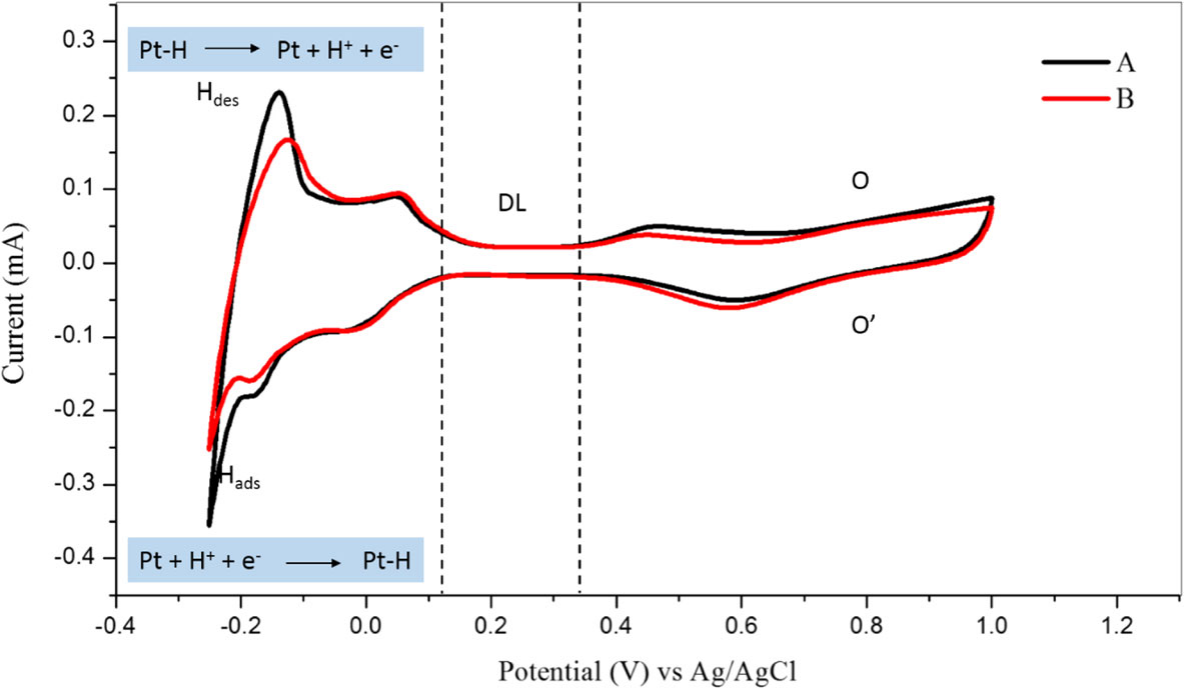
Cyclic voltammetry of [A] and [B] catalyst in 0.5 H_2_SO_4_. Scan rate: 50 mV/s. *A* Pt-Ru/TNT/C and *B* Pt/TNT/C

The electrochemical performance of the Pt-based electrode was tested by performing cyclic voltammetry with three-electrode configuration cell consisting of (i) a glassy carbon working electrode (*A* = 0.071 cm^2^), (ii) a Pt counter electrode and (iii) an Ag/AgCl reference electrode. In addition, 0.5 M H_2_SO_4_ and 1 M CH_3_OH were used as the supporting electrolyte for the methanol oxidation reaction investigation. The cyclic voltammetry of Pt-Ru/TNT/C and Pt/TNT/C electrocatalyst in 0.5 mol H_2_SO_4_ (acidic medium) is presented in Fig. 14. The CV curve shows there are three regions involved, as follows: (1) hydrogen adsorption and desorption (H_ads_, H_des_) from 0.12 to −0.25 V; (2) the double layer region (DL), where the residual current is capacitive current (i.e. the current becomes almost zero) from 0.12 to 0.35 V and (3) the oxygen region, which corresponds to the oxide formation from 0.7 to 1.0 V. In this region, O becomes Pt oxide during the oxidation at the metal surface by chemisorption of O species (OH_ads_ and O_ads_) and by oxygen reduction (O′) during the reverse scan. A well-defined peak was discovered at 0.58 V for both samples, corresponding to a desorption peak, which was attributed to O species. The cyclic voltammetry curve indicated that the Pt nanoparticles in the Pt-based electrodes were in good, stable electronic contact with the TNT surface because the metal was well dispersed onto the surface.

**Fig. 15:**
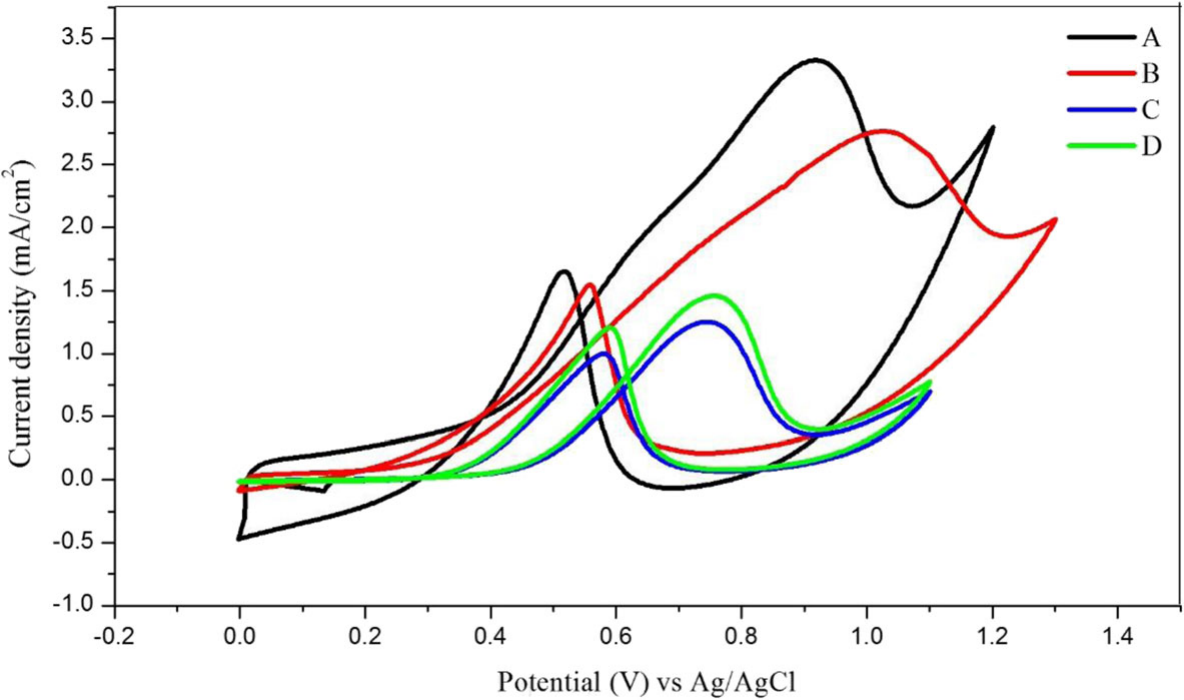
Current density from cyclic voltammetry of Pt-based catalyst in 1 M of CH_3_OH and 0.5 H_2_SO_4_. Scan rate: 50 mV; [A]: Pt-Ru/TNT/C, [B]: Pt/TNT/C, [C]: Pt-Ru/C, [D]: Pt/TiO_2_ nanoparticles. The current density was normalised by EASA of Pt and Pt-Ru

Figure 15 shows the cyclic voltammetry for the current density of the Pt-based catalyst in 0.5 M of H_2_SO_4_ and 1 M CH_3_OH scanned at 50 mV/s from 0 to 1.3 V. The current for all electrochemical analysis was normalised based on the electrochemically active surface area (EASA) of Pt and Pt-Ru. Sample [A], which is Pt-Ru/TNT-C, showed the highest current density for the oxidation peak, which was 3.30 mA/cm^2^ at 0.92 V, followed by sample [B], which is Pt/TNT/C, which was 2.77 mA/cm^2^ at 1.02 V. Both samples A and B showed higher current densities compared with those of samples C and D, which are Pt/Ru (1.25 mA/cm^2^) and Pt/TiO_2_ (1.46 mA/cm^2^) nanoparticles, respectively. This result demonstrates that the Pt-based catalyst supported on the TNTs gives outstanding performance for methanol oxidation based on the same catalyst loading of 10 wt%. For sample A, during the first scan, the onset potential for methanol oxidation was discovered at 0.2 V, and after that, a large anodic peak at 0.92 V on the positive irreversible scan and another acute peak indicating methanol oxidation at 0.52 V during the reverse scan were both observed. For sample B, the onset potential for methanol oxidation was discovered at 0.1 V, and a large anodic peak was observed at 1.02 V, corresponding, to methanol oxidation for the first sweep. During the reverse scan, an acute peak indicating the methanol oxidation reaction (MOR) was also discovered at 0.56 V. The second peak was discovered due to the oxidation of methanol and the removal of intermediate products, consisting of carbonaceous species (i.e. HCHO and HCOOH) that were generated during the incomplete MOR. The EASA of the Pt-based catalyst was calculated by measuring the charge from the hydrogen desorption peaks minus the charge from the double layer region, with assumption that the smooth Pt electrode gives the hydrogen adsorption a charge of 210 μC/cm^2^, which represents the quantity of charge corresponding to the adsorption of a monolayer of hydrogen on Pt. The EASA of Pt and Pt-Ru was calculated using the formula below:

8 $$ \mathrm{EASA}=\frac{Q_{\mathrm{H}}}{0.21\kern0.5em \mathrm{m}\mathrm{C}\cdotp {\mathrm{cm}}^{-2}x\ {M}_{\mathrm{cat}}}, $$

where *Q*_H_ is the charge due to the hydrogen adsorption/desorption in the hydrogen region of CV, 0.21 mC·cm^−2^ is the electrical charge associated with monolayer adsorption of hydrogen on Pt or Pt.Ru and *M*_cat_ is the loading of metal catalyst on the working electrode. The EASA of [A], Pt-Ru/TNT-C, has shown the highest value which is 0.79 cm^2^ than sample [B], Pt/TNT-C, which is 0.77 cm^2^. However, catalyst [C], Pt-Ru-C, shown higher EASA of 1.39 cm^2^ compared to [D], Pt/TiO_2_-C, which is 1.37 cm^2^. For TNT-based support catalyst (A and B), the EASA seem to be lower than non-TNT-based support catalyst (C and D) but the current density normalised by EASA calculated was higher than that non-TNT-based support catalyst. This can be explained that the improvement was due to the strong metal support interaction and better electronic conductivity of TNT itself which lead to decreasing the internal resistance of the methanol electro-oxidation reaction [4]. Therefore, the catalytic activity of A which consists of Pt-Ru/TNT-C was the best compared to another sample without TNT as catalyst support and got a lot improvement than previous studies [2, 5] reported.

**Fig. 16:**
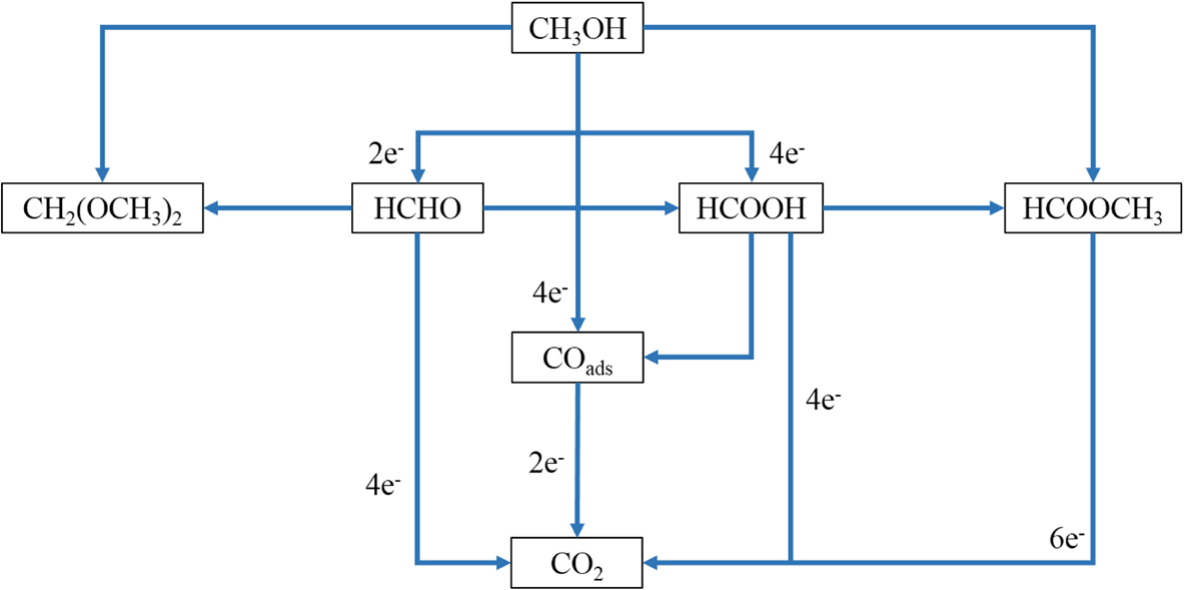
Possible pathway of generated CO species during methanol oxidation on Pt-Ru catalyst

The ratio of the forward oxidation peak current density (*j*_f_) to the reverse peak current density (*j*_r_), i.e. *j*_f_/*j*_r_, can be used to determine the catalyst’s tolerance to poisoning species. In methanol oxidation, the carbonaceous species HCHO and HCOOH may be generated when the methanol is incompletely electro-oxidized due to poor quantum yield that cause by the rapid recombination of photo-generated charges. Figure 16 shows the possible mechanism of intermediate species from carbonaceous species that may generated during methanol oxidation reaction via several possible pathways. The intermediate species that was formed during the incomplete methanol oxidation reaction was able to move on through the tubular structure of TNTs and was adsorbed to the Pt-Ru surface in which at the same time can act as self-poisons and can be oxidized again to CO_2_. Table 3 shows the characteristics of the Pt-based electrocatalysts and their electrochemical performance in the methanol oxidation reaction. The higher is the ratio of *j*_f_/*j*_r_, the more effective is the removal of poisoning species and the higher is the catalyst tolerance. The value of *j*_f_/*j*_r_ of [A] (Pt-Ru/TNT/C) showed the highest ratio of 2.0 among the electrocatalysts, which demonstrated that it was a better choice as the electrocatalyst for methanol oxidation as it showed better and higher tolerance towards carbonaceous species and decreased the poisoning effect. Table 4 shows the various comparisons of Pt-based catalyst for same application of methanol oxidation. From the table, we can see that the Pt-Ru/TNT-C catalyst in this study was in good agreement with previous studies. Although some improvements were needed, it is still comparable with other researchers. Equations 9–12 below show the general reaction of methanol oxidation and involving Pt-Ru catalyst. The synergetic effect of Pt-Ru alloy may be one of the reasons for improvement of CO poisoning as Ru was activated in the water molecules to form active oxidant and tend to help the oxidation of CO intermediate species on the Pt surface [33].

**Table 3:** Characteristics of Pt-based electrocatalyst and electrochemical performance

Sample	Electrode potential (V)	EASA (Pt or Pt-Ru)	Current density (mA/cm^2^), *j*_f_	Current density (mA/cm^2^), *j*_r_	*j*_f_/*j*_r_
A	0.92	0.79	3.30	1.66	2.0
B	1.02	0.77	2.77	1.55	1.79
C	0.74	1.39	1.25	0.99	1.26
D	0.75	1.37	1.46	1.21	1.21

Note: [A]: Pt-Ru/TNT/C, [B]: Pt/TNT/C, [C]: Pt-Ru/C, [D]: Pt/TiO_2_ nanoparticles

*j*_*f*_ forward oxidation peak of current density, *j*_*r*_ reverse oxidation peak of current density, *EASA* electrochemical active surface area

**Table 4:** Variation of Pt-based catalyst for methanol oxidation performance

Bil	Type of catalyst	Loading (wt%)	EASA of cat	*j*, (mA/cm^2^)	Mass activity, (mA/mg) of cat	Catalyst tolerance, *j*_f_/*j*_i_	Retention rates (%)	Ref
1	Pt-Ru/TNT-C	10	0.79 cm^2^	3.3	440 mA/mg	2.0	18	M. Abdullah
2	Pt/TNT	9.52	1.041 cm^2^	~1.65	–	1.04	–	[2]
3	Pt-HTN	10	–	3.0	–	–	–	[5]
4	Pt/C-HTNT	20	68.5 m^2^/g	–	530	–	22.6	[3]
5	Pt/C-TNT	20	89.7 m^2^/g		~400	–	–	[4]
6	Pt/C	12.4	2.23 cm^2^	~1.4	–	0.80	–	[2]
7	Pt/TiO_2_	1.83	0.28 cm^2^	~1.39	–	1.68	–	[2]
8	Pt/CNT	7.3	79 m^2^/g	–	–	0.89	–	[34]
9	Pt/TiO_2_	8.9	–	–	–	1.21	–	
10	Pt/CNT/TiO_2_	8.6	40 m^2^/g		–	0.99	–	

Note: The current density, *j*, and mass activity was normalised by EASA of catalyst used

9 $$ {\mathrm{CH}}_3\mathrm{O}\mathrm{H} + \mathrm{P}\mathrm{t}\to \mathrm{P}\mathrm{t}-\mathrm{C}\mathrm{O} + 4{\mathrm{H}}^{+} + 4{\mathrm{e}}^{-} $$

10 $$ \mathrm{R}\mathrm{u} + {\mathrm{H}}_2\mathrm{O}\to \mathrm{R}\mathrm{u}-\mathrm{O}\mathrm{H} + {\mathrm{H}}^{+} + {\mathrm{e}}^{-} $$

11 $$ \mathrm{T}\mathrm{i} + {\mathrm{H}}_2\mathrm{O}\to \mathrm{T}\mathrm{i}-\mathrm{O}\mathrm{H} + {\mathrm{H}}^{+} + {\mathrm{e}}^{-} $$

12 $$ 2\mathrm{P}\mathrm{t}-\mathrm{C}\mathrm{O} + \mathrm{R}\mathrm{u}-\mathrm{O}\mathrm{H} + \mathrm{T}\mathrm{i}-\mathrm{O}\mathrm{H}\to 2{\mathrm{CO}}_2 + 2\mathrm{P}\mathrm{t} + \mathrm{R}\mathrm{u} + \mathrm{T}\mathrm{i} + 2{\mathrm{H}}^{+} + 2{\mathrm{e}}^{-} $$

**Fig. 17:**
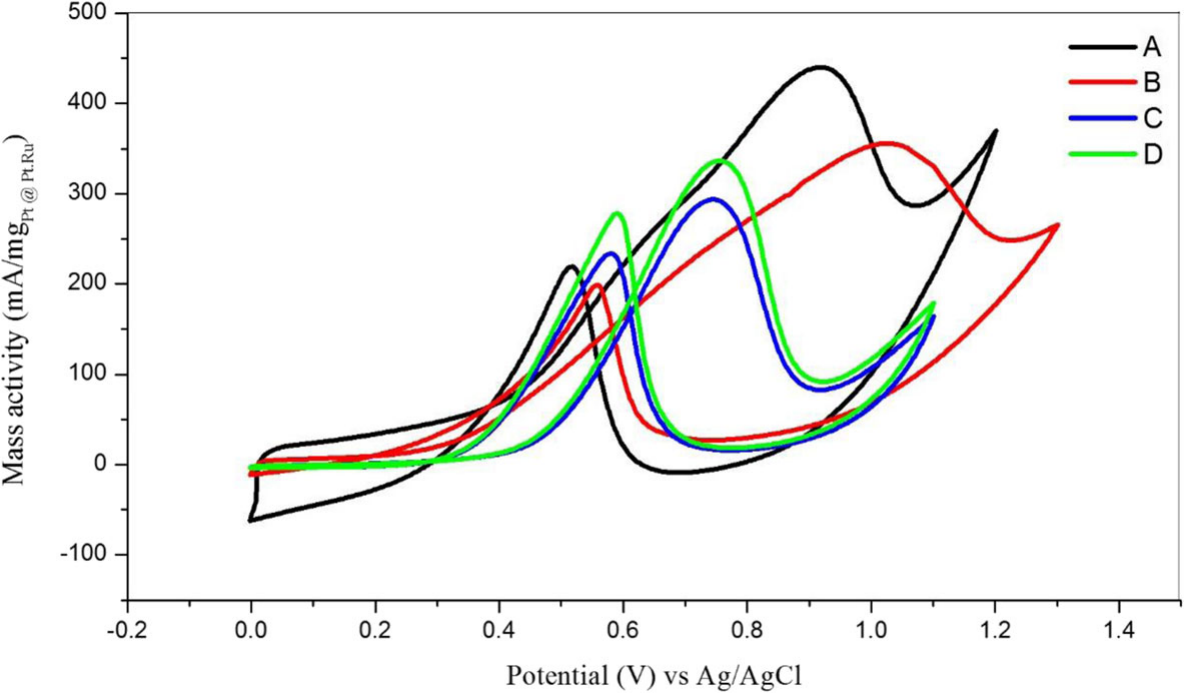
Mass activity of Pt-based catalyst from cyclic voltammetry in 1 M of CH_3_OH and 0.5 H_2_SO_4_. Scan rate: 50 mV/s. [A]: Pt-Ru/TNT/C, [B]: Pt/TNT/C, [C]: Pt-Ru/C, [D]: Pt/TiO_2_-C nanoparticles. The current density was normalised by EASA of Pt and Pt-Ru

Moreover, the Pt-Ru catalyst also play important role in achieving high electrocatalytic activity as it can easily dispersed onto the porous structure of TNTs. Figure 17 shows the cyclic voltammetry of mass activity for all Pt-based electrocatalysts in the acidic methanol medium. The curves show that the mass activities of samples [A], [B], [C] and [D] are 440, 356, 294 and 337 mA/mg of catalyst, respectively. Pt-Ru/TNT/C showed the highest activity with 440 mA/mg of the Pt-Ru catalyst compared with the other catalysts. Moreover, the Pt-Ru catalyst supported by TNT-C from this study also showed great improvement compared to previous study done by higher loading of catalyst for methanol oxidation reaction of the same catalyst type [6, 34] and with same TNT as support [2–5]. This result indicated that the reaction internal resistance for A was relatively smaller than that of the other electrocatalyst [3], and A can be considered the most effective structure for a strong metal support interaction [23]. Furthermore, sample D consists of Pt/TiO_2_-C also has shown higher mass activity than sample C which does not contain TiO_2_ as a support. This result also suggested that the combination of TiO_2_ with C can also help to promote the methanol oxidation by providing space for dispersion of catalyst and of course with the nanotubes structures of TiO_2_, gave the most promising result as the surface area of TNT was higher than that of nanoparticles of TiO_2_.

### Chronoamperometric

**Fig. 18:**
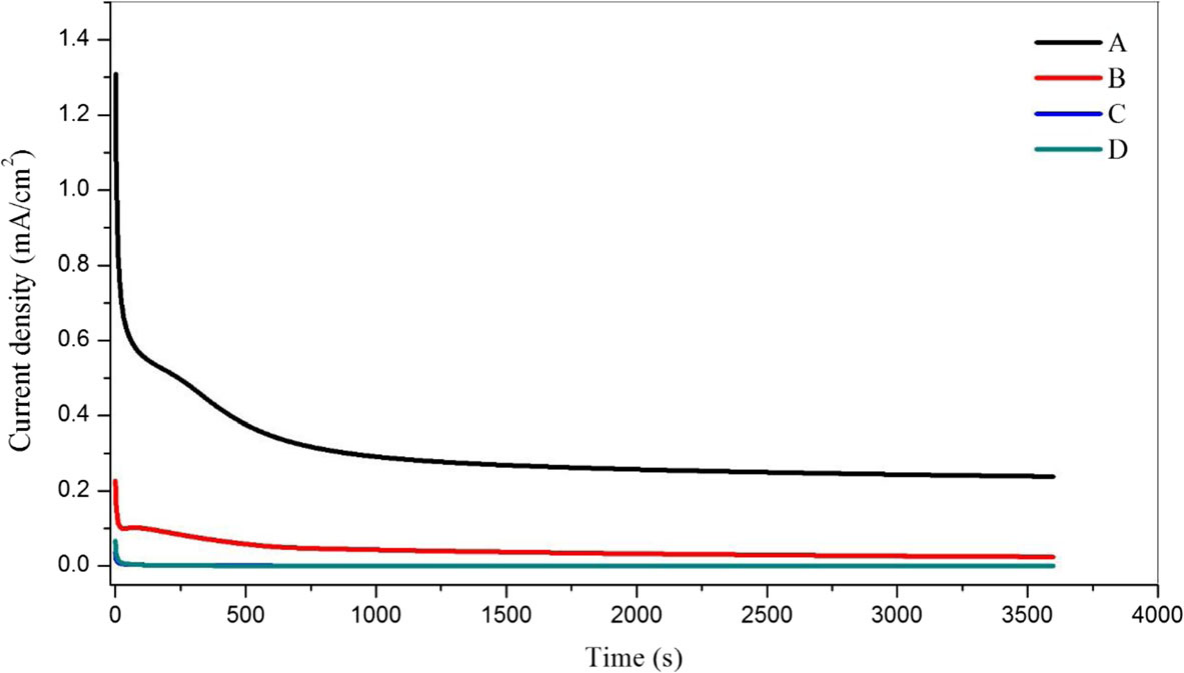
Chronoamperometric curves for Pt-based catalyst in methanol acidic medium. Potential 0.5 V, time 3600 s. The current density was normalised by EASA of Pt and Pt-Ru

The performance in terms of stability of the electrode during methanol oxidation was examined by chronoamperometric analysis. Figure 18 shows the CA curves for Pt-Ru/TNT/C, Pt/TNT/C, Pt/TiO_2_/C and Pt/Ru/C, which are labelled as [A], [B], [C] and [D], respectively, at 0.5 V for 3600 s in 0.5 M H_2_SO_4_ and 1 M CH_3_OH. The stability of catalysts can be examined by the ratio of the final current density, *j*_f_, to the initial current, *j*_i_, (i.e. *j*_f_/*j*_i_) obtained for each type of catalyst. Table 5 presents the values of the initial and final current densities for samples A, B, C and D that was normalised by each EASA. The final current densities of A, B, C and D are 0.24, 0.024, 1.33 × 10^−4^ and 2.3 × 10^−4^ mA/cm^2^, respectively. The relative ratios of the current densities for A, B, C and D are 0.18, 0.10, 0.0037 and 0.0034, respectively. These results indicate that sample A, which is Pt-Ru/TNT/C, shows the best activity and stability with highest retention rates of 18 % as the catalyst for methanol oxidation compared with the other electrocatalysts for over than 3600 s. Moreover, the result also in agreement with previous study achieved [3].

**Table 5:** Retention rates from chronoamperometric curves

Sample	*j*_i_ (mA/cm^2^)	*j*_f_ (mA/cm^2^)	Ratio *j*_f_/*j*_i_	Retention rates (%)
A	1.31	0.24	0.18	18
B	0.23	0.024	0.10	10
C	0.036	1.33 × 10^−4^	0.0037	0.37
D	0.068	2.3 × 10^−4^	0.0034	0.34

[A]: Pt-Ru/TNT/C, [B]: Pt/TNT/C, [C]: Pt-Ru/C, [D]: Pt/TiO_2_ nanoparticles. The current density was normalised by EASA of Pt and Pt-Ru

*j*_i_ initial current density, *j*_f_ final current density

## Conclusions

In this study, TNTs were successfully fabricated using a hydrothermal method at 180 °C for 48 h by varying the concentration of highly concentrated NaOH. The effect of the NaOH concentration was thoroughly studied, and the results imply that TNT-10 has the best properties out of all the TNTs, with the complete formation of nanotubes providing the highest surface area. TNT-10 was then used as the support for Pt-based catalysts, and it showed high electrocatalytic activity for methanol oxidation. The use of TNTs as a catalyst support for Pt-based catalysts could enhance the electrocatalytic performance by enabling excellent dispersion of the catalyst onto the high specific surface of TNTs. The final results suggest that the Pt/Ru/TNT/C catalyst shows high performance in methanol oxidation by reducing the poisoning effect of generated CO species and by demonstrating high retention rates and stability for over than 3600 s.

## References

[1] Tamašauskaitė-Tamašiūnaitė L, Balčiūnaitė A, Vaiciukevičienė A, Selskis A, Norkus E (2013). Investigation of electrocatalytic activity of titania nanotube supported nanostructured Pt–Ni catalyst towards methanol oxidation. J Power Sources.

[2] Abida B, Chirchi L, Baranton S, Napporn TW, Kochkar H, Léger J-M (2011). Preparation and characterization of Pt/TiO2 nanotubes catalyst for methanol electro-oxidation. Appl Catal Environ.

[3] Sui X-L, Wang Z-B, Li C-Z, Zhang J-J, Zhao L, Gu D-M (2014). Effect of pH value on H2Ti2O5/TiO2 composite nanotubes as Pt catalyst support for methanol oxidation. J Power Sources.

[4] Sui X-L, Wang Z-B, Yang M, Huo L, Gu D-M, Yin G-P (2014). Investigation on C–TiO2 nanotubes composite as Pt catalyst support for methanol electrooxidation. J Power Sources.

[5] Abida B, Chirchi L, Baranton S, Napporn TW, Morais C, Léger J-M (2013). Hydrogenotitanates nanotubes supported platinum anode for direct methanol fuel cell. J Power Sources.

[6] Feng C, Takeuchi T, Abdelkareem MA, Tsujiguchi T, Nakagawa N (2013). Carbon–CeO2 composite nanofibers as a promising support for a PtRu anode catalyst in a direct methanol fuel cell. J Power Sources.

[7] Hosseini MG, Momeni MM (2012). UV-cleaning properties of Pt nanoparticle-decorated titania nanotubes in the electro-oxidation of methanol: an anti-poisoning and refreshable electrode. Electrochim Acta.

[8] Yu S, Liu Q, Yang W, Han K, Wang Z, Zhu H (2013). Graphene–CeO2 hybrid support for Pt nanoparticles as potential electrocatalyst for direct methanol fuel cells. Electrochim Acta.

[9] Xing L, Jia J, Wang Y, Zhang B, Dong S (2010). Pt modified TiO2 nanotubes electrode: preparation and electrocatalytic application for methanol oxidation. Int J Hydrogen Energy.

[10] Georgieva J, Sotiropoulos S, Valova E, Armyanov S, Karanasios N (2014). Methanol oxidation and photo-oxidation at Pt/WO3 electrocatalysts on graphite substrates. J Electroanal Chem.

[11] Wang H, Wang X, Zheng J, Peng F, Yu H (2014). Pt/MoO3-WO3/CNTs catalyst with excellent performance for methanol electrooxidation. Chin J Catalysis.

[12] Wang T, Tang J, Wu S, Fan X, He J (2014). Preparation of ordered mesoporous WO3–TiO2 films and their performance as functional Pt supports for synergistic photo-electrocatalytic methanol oxidation. J Power Sources.

[13] Yang C, van der Laak NK, Chan K-Y, Zhang X (2012). Microwave-assisted microemulsion synthesis of carbon supported Pt-WO3 nanoparticles as an electrocatalyst for methanol oxidation. Electrochim Acta.

[14] Cao H, Wang Z, Hou G, Zheng G (2010). TiO2 nanotube-supported amorphous Ni–B electrode for electrocatalytic oxidation of methanol. Surf Coat Technol.

[15] Ju J, Chen X, Shi Y, Wu D (2013). A novel PdAg/TiO2 nanotube electrocatalyst for methanol electro-oxidation. Fuel.

[16] Santara B, Imakita K, Fujii M, Giri PK (2016). Mechanism of defect induced ferromagnetism in undoped and Cr doped TiO2 nanorods/nanoribbons. J Alloys Compd.

[17] Bai H, Liu L, Liu Z, Sun DD (2013). Hierarchical 3D dendritic TiO2 nanospheres building with ultralong 1D nanoribbon/wires for high performance concurrent photocatalytic membrane water purification. Water Res.

[18] Zhu L, Cao L, Su G, Liu W, Song L, Liu H (2011). Effect of post heat treatment on microstructure and photocatalytic activities of TiO2 nanoribbons. Appl Surf Sci.

[19] Wong CL, Tan YN, Mohamed AR (2011). A review on the formation of titania nanotube photocatalysts by hydrothermal treatment. J Environ Manage.

[20] Tang Y, Liu L, Wang X, Jia D, Xia W, Zhao Z (2016). TiO2 quantum dots embedded in bamboo-like porous carbon nanotubes as ultra high power and long life anodes for lithium ion batteries. J Power Sources.

[21] Kasuga T (2006). Formation of titanium oxide nanotubes using chemical treatments and their characteristic properties. Thin Solid Films.

[22] Kasuga T, Hiramatsu M, Hoson A, Sekino T, Niihara K (1998). Formation of titanium oxide nanotube. Langmuir.

[23] Ito Y, Takeuchi T, Tsujiguchi T, Abdelkareem MA, Nakagawa N (2013). Ultrahigh methanol electro-oxidation activity of PtRu nanoparticles prepared on TiO2-embedded carbon nanofiber support. J Power Sources.

[24] Huang J, Cao Y, Deng Z, Tong H (2011). Formation of titanate nanostructures under different NaOH concentration and their application in wastewater treatment. J Solid State Chem.

[25] Cui L, Hui KN, Hui KS, Lee SK, Zhou W, Wan ZP (2012). Facile microwave-assisted hydrothermal synthesis of TiO2 nanotubes. Mater Lett.

[26] Liu W, Sun W, Han Y, Ahmad M, Ni J (2014). Adsorption of Cu(II) and Cd(II) on titanate nanomaterials synthesized via hydrothermal method under different NaOH concentrations: role of sodium content. Colloids Surf A Physicochem Eng Asp.

[27] I. Tacchini AA-C, Youhai Yu, M.T. Martinez, M. Lira-Cantu. Hydrothermal synthesis of 1D TiO2 nanostructures for dye sensitized solar cells.

[28] Dong B, He B, Chai YM, Liu CG (2010). Novel Pt nanoclusters/titanium dioxide nanotubes composites for hydrazine oxidation. Mater Chem Physics.

[29] Dong B, He B-L, Huang J, Gao G-Y, Yang Z, Li H-L (2008). High dispersion and electrocatalytic activity of Pd/titanium dioxide nanotubes catalysts for hydrazine oxidation. J Power Sources.

[30] Hai-chao Liang X-zL, Janusz Nowotny. Photocatalytic properties of TiO2 nanotubes.

[31] Camposeco R, Castillo S, Mejia-Centeno I, Navarrete J, Gómez R (2014). Effect of the Ti/Na molar ratio on the acidity and the structure of TiO2 nanostructures: nanotubes, nanofibers and nanowires. Mater Characterization.

[32] Lee D-S, Lee S-Y, Rhee KY, Park S-J (2014). Effect of hydrothermal temperature on photocatalytic properties of TiO2 nanotubes. Current Applied Physics.

[33] Ju J, Shi Y, Wu D (2012). TiO2 nanotube supported PdNi catalyst for methanol electro-oxidation. Powder Technol.

[34] Kim M-S, Fang B, Chaudhari NK, Song M, Bae T-S, Yu J-S (2010). A highly efficient synthesis approach of supported Pt-Ru catalyst for direct methanol fuel cell. Electrochim Acta.

